# Ultra-Rapid Laser Calorimetry for the Assessment of Crystallization in Low-Concentration Cryoprotectants

**DOI:** 10.1115/1.4052568

**Published:** 2022-02-07

**Authors:** Joseph Kangas, Li Zhan, Yilin Liu, Harishankar Natesan, Kanav Khosla, John Bischof

**Affiliations:** Department of Mechanical Engineering, University of Minnesota-Twin Cities, Minneapolis, MN 55408; Department of Mechanical Engineering, University of Minnesota-Twin Cities, Minneapolis, MN 55408; Department of Biomedical Engineering, University of Minnesota-Twin Cities, Minneapolis, MN 55408

**Keywords:** calorimetry, laser calorimetry, cryoprotectant, critical cooling rate, critical warming rate, vitrification

## Abstract

Cryoprotective agents (CPAs) are routinely used to vitrify, attain an amorphous glass state void of crystallization, and thereby cryopreserve biomaterials. Two vital characteristics of a CPA-loaded system are the critical cooling and warming rates (CCR and CWR), the temperature rates needed to achieve and return from a vitrified state, respectively. Due to the toxicity associated with CPAs, it is often desirable to use the lowest concentrations possible, driving up CWR and making it increasingly difficult to measure. This paper describes a novel method for assessing CWR between the 0.4 × 10^5^ and 10^7^ °C/min in microliter CPA-loaded droplet systems with a new ultrarapid laser calorimetric approach. Cooling was achieved by direct quenching in liquid nitrogen, while warming was achieved by the irradiation of plasmonic gold nanoparticle-loaded vitrified droplets by a high-power 1064 nm millisecond pulsed laser. We assume “apparent” vitrification is achieved provided ice is not visually apparent (i.e., opacity) upon imaging with a camera (CCR) during cooling or highspeed camera (CWR) during warming. Using this approach, we were able to investigate CWRs in single CPA systems such as propylene glycol (PG), glycerol, and Trehalose in water, as well as mixtures of glycerol-trehalose-water and propylene glycol-trehalose-water CPA at low concentrations (20–40 wt %). Further, a phenomenological model for determining the CCRs and CWRs of CPAs was developed which allowed for predictions of CCR or CWR of single component CPA and mixtures (within and outside of the regime their constituents were measured in), providing an avenue for optimizing CCR and CWR and perhaps future CPA cocktail discovery.

## 1 Introduction

Cryopreservation allows for long-term storage because the chemical activity normally associated with functioning cells effectively ceases at low cryogenic storage temperatures such as at the boiling point of liquid nitrogen, or −196 °C. In fact, the only source of damage to biological material at this temperature is from direct ionization from background radiation, making storage durations of the order of millennia possible [1]. Under normal circumstances, cooling biological materials to these temperatures leads to widespread cell death due to the damaging effects of ice crystallization; however, cryoprotective agents (CPAs) can be introduced to mitigate some of these effects. These CPAs work to preserve biomaterials by modulating the viscosity, glass and melting transition temperatures, and other physical properties to limit the scale and scope of ice formation [2–4]. Ice formation, both intracellular and extracellular, can lead to cellular damage and death, with intracellular ice formation linked directly to cell membrane damage [5–8]. However, many CPAs are not well tolerated by cells, and so proper cryopreservation requires achieving a delicate balance between the CPA toxicity and the damage caused by ice formation. Toxic effects have been shown to occur at different concentrations for different CPAs, and these effects also vary widely between cell and tissue types [7,9]. In general, high-concentration CPAs exhibit strong ice-suppressing properties but unfortunately have been shown to be more cytotoxic than low-concentration CPAs and are capable of damaging or destroying cells via osmotic affects [10,11]. This limitation suggests that it is important to use the lowest possible concentration of CPA to prevent toxicity to the biological system, which thereby necessitates high rates of cooling and warming. For microliter systems, one common approach is to bring the system to a vitrified state, transforming the biomaterial into an amorphous glass while eliminating the damage caused by ice crystal formation and its accompanying osmotic shock. Slow freezing is an alternative approach to systems at this scale, whereby cooling slowly, ice is restricted to the extracellular space; however, this is often accompanied by larger osmotic gradients and volume changes versus vitrification and will not be further pursued here [12].

The successful vitrification of biological systems in microliter-sized droplets is dependent on the concentration of CPA used, with each concentration having a corresponding critical cooling rate (CCR) needed to achieve vitrification on cooling and critical warming rate (CWR) to avoid devitrification on warming. To limit the toxic effects of CPAs while also mitigating ice formation at the cooling rates (∼10^4^ °C/min) experienced at this scale, microliter-scale cryopreservation requires CPA concentrations of roughly 30 wt %, which can vary by as much as 10 wt % depending on the CPA used. The CWRs at these low concentrations can be several orders of magnitude larger than the CCRs, requiring warming rates in excess of 10^6^ °C/min. This difference largely occurs because the temperature at which peak crystal growth occurs is near the melting temperature, whereas the temperature of peak nucleation occurs well below the melting point. This means that during cooling, one passes through the temperature window in which crystal growth is maximal before much nucleation has occurred, whereas, during warming, the majority of nuclei form before the period of maximal crystal growth, causing much more ice to form relative to cooling at the same rate. Additionally, to measure the CWR, one must first cool the sample down. This inherently leads to some crystallization and nucleation, effectively seeding and boosting ice growth once warming commences and thereby inflating the CWR measurement. The current practice is to minimize this affect by cooling at rates far above the CCR, but this practice works in only the high-concentration regime. Note that the elimination of all ice crystallization during cooling or warming is impossible without infinite temperature rates or infinite time, and in the absence of either of these, some fraction of the sample will crystalize regardless of the cooling or warming rate [13–16]. Thus, instead of using ideal vitrification as a marker for success, it is often more practical to use “apparent vitrification.” An ice fraction threshold commonly used to define apparent vitrification is less than 0.2% ice by mass [17]. Throughout the remainder of this paper, the use of the word *vitrification* implies *apparent vitrification.* Understanding the relationship between the cryoprotectant concentration and ice formation is paramount for successful cryopreservation and rewarming.

Currently, determining the necessary CCRs and CWRs of CPAs, especially in the low-concentration regime, remains a challenge, with a summary of the current techniques in Table 1. For instance, conventional CCR and CWR measurements have relied on DSC, but conventional machines can only attain rates up to roughly 100 °C/min in the cryogenic range, though DSC has the advantage of quantitative ice detection [18–20]. A promising new technology, termed nanocalorimetry, being developed at several institutions including the National Institute of Standards and Technology may allow DSC to be carried out at millions of degrees per minute for extremely small samples [21–23]. Unfortunately, to get rates of millions of degrees per minute requires samples on the order of a picoliter, which without special care, will quickly evaporate (especially for CPAs containing volatiles like alcohols), necessitating precise environmental control to measure CWR of CPAs with nanocalorimetry above 10^6^ °C/min [24,25]. The volume dependence of nucleation in water droplets near the homogeneous nucleation temperature, primarily due to surface nucleation effects on the droplet, has been shown to become significant for droplet diameters less than 50 *μ*m and to dominate for diameters of less than 10 *μ*m [26]. This means even if the barriers to evaporation are overcome for nanocalorimetry, droplets of less than 100 pL will experience appreciable surface nucleation, thus any CWR measurements will not be meaningful when applied to larger systems. It remains unclear the total contribution to crystallization surface nucleation has when cooling over a large temperature regime, as well as in CPAs.

**Table 1:** Current methods for analyzing CWR with rates, benefits, and drawbacks

	Conventional differential scanning calorimetry (DSC)	Convective warming	Nano-DSC	Laser calorimetry
Cooling rates (C/min)	0.1–50	10^3^–10^5^	10^3^–10^7^	10^3^–10^5^
Warming rates (C/min)	0.1–100	10^3^–10^6^	10^3^–10^7^	10^2^–10^8+^
Sample size	1–10 *μ*L	1 nL–100 *μ*L	1 pL–1 nL	1 nL–100 *μ*L
Benefits	Highly sensitive. Quantitative phase change detection.	Low cost. Simple implementation.	Highly sensitive. Quantitative phase change detection. Large rate regime.	Large rate regime and arbitrarily large warming rates. Volumetric warming.
Drawbacks	Slow rates, especially on cooling. Difficult to maintain cooling rates in the cryogenic temperature range.	Volume limited rates. Qualitative ice detection.	Expensive. Evaporation. Appreciable surface nucleation.	Qualitative ice detection. Limited by the achievable cooling rate.

An additional method used to measure CCR and CWR is by directly quenching a droplet with a thermocouple inside it into liquid nitrogen for cooling or hot oil for warming, then imaging the subsequent process to determine if ice formation occurred. CWRs of up to 10^6^ °C/min have been measured via direct quenching into hot oil [27]. CCR on the other hand has not been directly measured much above 10^5^ °C/min due to a combination of factors making the achievable rates lower than convective warming. First, convective cooling involves quenching into a cold fluid, typically liquid nitrogen, so the maximum temperature difference between the bath and sample is about 200 °C whereas convective warming can have temperature differences of 400 °C or larger using hot oil. Additionally, quenching into liquid nitrogen introduces the Leidenfrost effect, where upon quenching a thin layer of liquid nitrogen vapor blankets the sample and reduces heat transfer, and thus cooling rate [28]. Depending on the size and temperature of the sample being plunged heat transfer coefficients for liquid nitrogen vary from less than 1000 W/(m^2^ °C) up to 10^6^ W/(m^2^ °C) [29,30]. Measuring CWRs above 10^5^ °C/min requires nanoliter-scale droplet volumes, which in turn require extremely fine-gage thermocouples to measure the warming rate without introducing uncertainty due to the thermal mass of the thermocouple. Due to these problems, CWRs in excess of 10^6^ °C/min have yet to be measured; however, with the advent of the laser nanowarming of small specimens (cells, embryos, and larvae) in dilute CPAs, calorimetric data in this regime are becoming increasingly important [9,31,32]. The use of a high-power laser and highly absorbent nanoparticles has been demonstrated to attain uniform warming rates in excess of 10^7^ °C/min in microliter-scale droplets, providing an excellent framework upon which an ultrarapid laser calorimeter can be used to detect the CWRs of cryoprotectants in regimes previously unattainable, thereby allowing for the decoupling of cooling rate and CWR measurements [32].

**Fig. 1:**
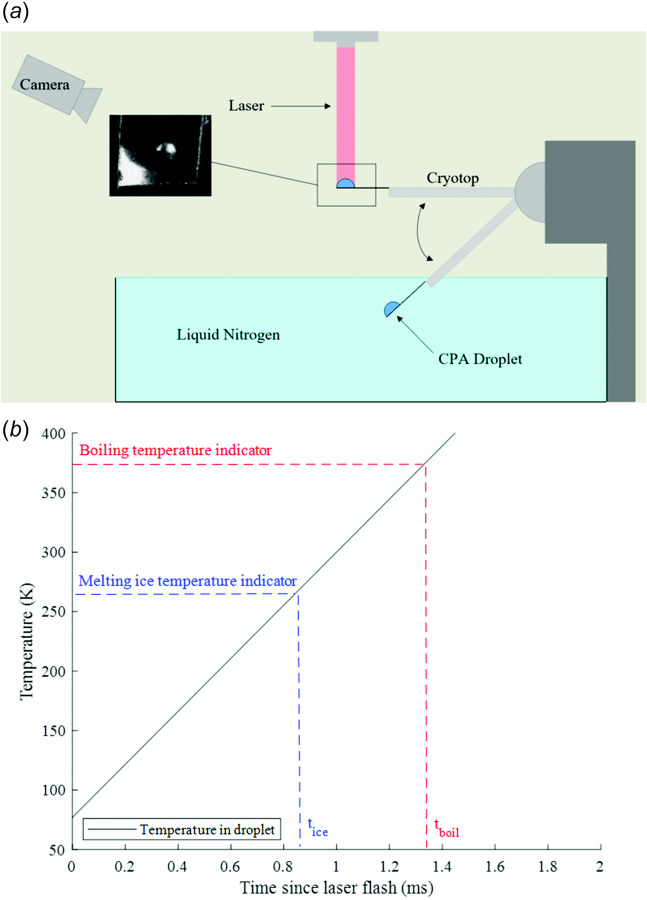
(*a*) Schematic of laser warming and (*b*) plot showing how warming rates are calculated from temperature indicators in high-speed videos of laser warming

In this study, a high-power pulsed laser, high-speed camera, and gold nanoparticles (GNPs) were used to create a laser calorimeter (Fig. 1) to measure the CWRs of low concentrations of propylene glycol (PG), trehalose, and glycerol and mixtures of glycerol and PG with trehalose in the 10^6^–10^7^ °C/min warming rate range (20–40 wt %). CCRs were also measured via direct quenching to determine how the relationship between CCR and CWR changes with CPA concentration. In addition to these experiments, a theory for determining the CCR and CWR of mixtures of CPAs was developed and tested. To help understand the thermal gradients and warming rates measured by laser calorimetry, Monte Carlo (MC) simulations of photon transport and finite element heat transfer modeling were carried out.

## 2 Model Development

A vast amount of experimental evidence indicates that the CCR and CWR of a CPA have an exponential relationship with concentration [33,34]. By taking advantage of this property, we can create a model that describes what happens to CCR or CWR of mixtures of CPAs, given knowledge of the CPAs in the mixture. For brevity, only the CCR will be considered in the following analysis, though it is also valid for CWR as well.

### 2.1 Single Species Critical Cooling Rate Estimation.

Since CCR depends exponentially on concentration, it can be expressed in the form

(1) $$R=Ae^{-\alpha c}$$

where A and α are constants, c is the weight fraction concentration, and R is the CCR. In principle, the CCRs of all CPAs should have identical A values since at zero concentration they are all identically water. Consider a mixture of two CPAs at concentrations $c_{1}$ and $c_{2}$ whose individual CCRs can be expressed by the equations

(2) $$R_{1}=Ae^{-\alpha_{1}c_{1}},\;R_{2}=Ae^{-\alpha_{2}c_{2}}$$

### 2.2 Two-Species Critical Cooling Rate Estimation.

The CCR of the mixture of these two CPAs must take the form of $R_{1,2}$ (Eq. (3)), which is further discussed in the Supplemental Materials. This reduces to $\;R_{1}$ and $R_{2}$ for $c_{2}=0$ and $c_{1}=0$ , respectively, where f is some unknown function.

(3) $$R_{1,2}=Ae^{-\alpha_{1}c_{1}}e^{-\alpha_{2}c_{2}}e^{-{f(\alpha }_{1},\alpha_{2},c_{1},c_{2}){c_{1}c}_{2}}$$

The function f cannot be determined a priori; however, experimental evidence may provide insights into its functional form. The relationship between exponential factors, shown in Fig. S8 available in the Supplemental Materials on the ASME Digital Collection for the CCR, was determined to be a linear average of their respective concentrations. Since the exponential factors of a unary trehalose solution and a unary glycerol solution are almost identical, the exponential factor of glycerol-trehalose mixtures has almost no concentration dependence. Using this linear relationship, we arrive at a functional form for f and thus an expression for $R_{1,2}$

(4) $$f=\frac{\left(\alpha_{2}-\alpha_{1}\right)}{\chi_{1}}$$

(5) $$R_{1,2}=Ae^{-\alpha_{1}c_{1}}e^{-\alpha_{2}c_{2}}e^{-\frac{\alpha_{2}-\alpha_{1}}{\chi_{1}}c_{1}c_{2}}$$

**Fig. 2:**
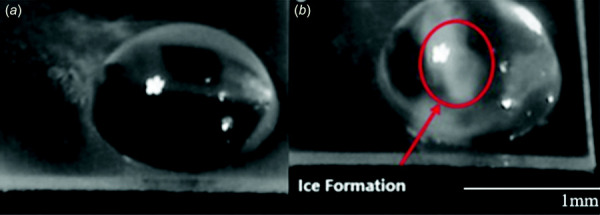
The figure above shows a vitrified droplet (*a*) and a droplet midpulse during laser warming (*b*). The opaque areas in the middle of the droplet on the right correspond to ice formation during warming, indicating that the warming rate was lower than the CWR. The opaque areas in the vitrified droplet on the left are actually reflected images of the laser chamber interior. One characteristic of the nucleated ice is that it moves within the droplet during laser warming (see videos of laser warming in the Supplemental Materials), providing a distinction between these artifacts and ice.

**Fig. 3:**
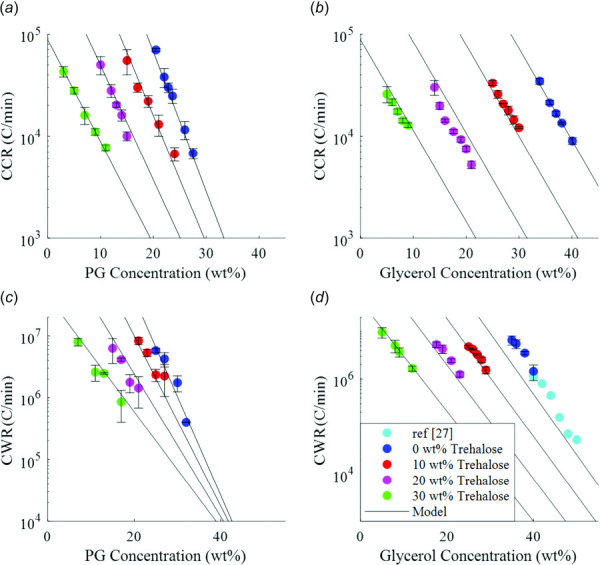
Measured CCRs and CWRs of CPA solutions. Plots show CCR of (*a*) PG-trehalose solutions and (*b*) glycerol-trehalose solutions; and CWRs of (*c*) PG-trehalose solutions and (*d*) glycerol-trehalose solutions. Each plot includes the two-species model (Eq. (6)) for the CCR/CWR based on the CCR/CWR measurements of single-species solutions of trehalose, PG, and glycerol [27].

In this equation, $\chi_{1}$ is the concentration weight fraction for species 1 at saturation in water. For trehalose, this value is roughly 0.4, corresponding to solubility of 69 g per 100 g of water.

### 2.3 Multispecies Critical Cooling Rate Estimation.

From this expression, we can apply the same argument for a mixture of n CPAs. Additional discussion for when the leading pre-exponential constant *A* varies between CPAs can be found in the Supplemental Materials on the ASME Digital Collection

(6) $$R_{n}({c_{1},\ldots ,c}_{n})=A\prod_{i=1}^{n}e^{\left(-\alpha_{i}c_{i}\right)}\prod_{1\leq i<j\leq n}e^{-\frac{\alpha_{j}-\alpha_{i}}{\chi_{i}}c_{i}c_{j}}$$

In summary, Eq. (6) can be applied to both CCR and CWR experimental results in single and mixture CPA solutions.

## 3 Methods

### 3.1 Critical Cooling Rate Measurements.

The protocol for measuring CCR and identifying ice versus vitrification can be found in Sec. S1, available in the Supplemental Materials.

### 3.2 Laser Calorimetry.

The experimental setup for the laser calorimetry system can be seen in Fig. 1. Microliter droplets (0.1–0.5 *μ*L) of a CPA and gold nanorod (GNR) solution were placed on the tip of a cryotop and plunged into LN_2_. Droplet solutions were chosen so that the cooling rate of plunging was sufficiently above the CCR of the CPA to minimize crystallization during cooling. Next, a 2-mm-diameter unpolarized 1064 nm pulse laser with a peak power of 10 kW (LaserStar, Model Number 585-986-080) was fired vertically on the top of the hemispherical droplet, initiating plasmonic heating facilitated by the presence of GNRs, allowing for ultrarapid warming of the droplet. The concentration-dependent absorption coefficient for the GNR solution was measured via UV–Vis spectroscopy, see Fig. S9 available in the Supplemental Materials. From this data, the GNR concentration (1.65 × 10^16^ –6.83 × 10^16^ nps/m^3^) was chosen so that the absorption coefficient was between 0.5 cm^−1^ and 2.0 cm^−1^, corresponding to a transmittance of 80–95% through the microliter-(mm) scale droplets assuming Beer's law, allowing for warming rates between 4.0 × 10^5^ and 10^7^ °C/min given the energy of the laser [32,36]. Since the transmittance was large, there was negligible attenuation of the laser throughout the droplet, thus leading to relatively uniform warming rates. During warming, a high-speed camera (MEMRECAM Qv1) with an adjustable lens (Nikon ED 200 mm 1:4D) was used to record the changes in the droplet. Frame rates between 4000 and 15,000 frames per second (fps) were used over the course of this study. The maximum frame rate of the camera is 84,000 fps, which corresponds to a maximum detectable rate of the order of 10^8^ °C/min, assuming 10 frames are needed to resolve the warming rate. Dynamic ice detection was performed by visually identifying the occurrence of ice (i.e., a white opaque regions) in droplets using high-speed video footage. Image processing tools that adjusted the contrast aided in the detection of ice near the visual limit of detection. Figure 2 shows the difference between a vitrified droplet and one with ice during warming. Due to the high-speed camera's ability to detect some near-infrared light, there was a bright flash at the moment the laser fired. This flash allowed for the synchronization of the high-speed camera and laser pulse, thus determining the exact time the laser fired.

**Fig. 4:**
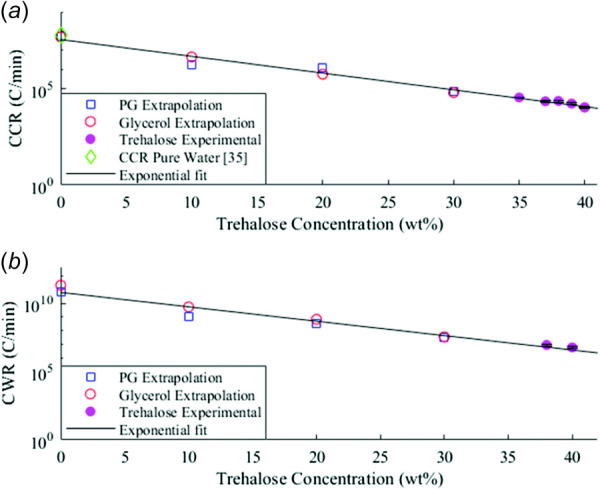
(*a*) CCRs of trehalose gathered by direct measurement and extrapolation of the CCRs from glycerol-trehalose and PG-trehalose solutions. Also shown is the predicted CCR of pure water. (*b*) CWRs of trehalose were gathered via laser calorimetry of trehalose solutions and extrapolations from the laser calorimetry of glycerol-trehalose and PG-trehalose solutions [35].

The CWR of the CPA was determined by modulating the laser energy in the following fashion. First, the droplet was warmed at the lowest laser setting (4.92 × 10^7^ W/m^3^) and assessed for ice. If no ice was detected, the concentration of GNRs in the droplet was decreased until a concentration that led to ice formation was identified. Once a sufficiently low GNR concentration that led to ice formation was identified, the laser voltage was increased by increments of 5 V, which increased the warming rate slightly. This procedure of increasing the laser voltage was continued until no ice was detected during warming. The warming rate was observed when any further increase in laser voltage led to no detectable ice was identified as the CWR. The warming rates were attained through analysis of the high-speed video, from which the times at which the laser fired, ice formed, ice completely melted, and the sample boiled were determined. By assuming that the temperatures of the droplet when the ice melted and liquid boiled are the melting and boiling temperatures of the CPA solution, respectively, and that the initial temperature of the droplet is −196 °C (LN_2_ temperature), the high-speed camera data can be used to arrive at two different values for the average warming rate in the droplet. The laser was fired just before the residual liquid nitrogen evaporating, so we are confident that the initial temperature of the droplet is −196 °C. The internal droplet temperature after leaving the LN_2_ bath was measured and showed the droplet temperature remains at LN_2_ temperature until the boiling of adjacent LN_2_ has ceased. Since the laser was fired once the residual LN_2_ evaporated, we are confident in the assumption that the droplet is at LN_2_ temperature the moment the laser fires. The final warming rate was determined by averaging the warming rates estimated based on the temperature indicators of ice melting and liquid boiling, assuming ice was detected. In the cases where no ice was detected, the boiling temperature indicator was used alone. These scenarios allowed for more accurate upper bound error estimation. Figure S11 available in the Supplemental Materials on the ASME Digital Collection shows high-speed videos of under-warmed and critically warmed droplets.

## 4 Results and Discussion

### 4.1 Validity of Visual Assay to Determine Critical Cooling Rate and Critical Warming Rate.

Visual detection of melting and boiling is key for this analysis to be valid. By warming below the CWR we are able to see ice form and then subsequently melt as the droplet temperature increases. The time at which all ice has melted (disappeared) is noted and corresponds to the droplet being at the melting temperature. Thus, by using the frame data we can then calculate the warming rate since we know the time at two temperature points (LN2 and melting). We slowly increasing the warming rate until no ice was formed on warming. This was the critical warming rate calculated from ice melting. For boiling, we used pulses that were longer than necessary to bring the droplet up to room temperature, thus causing the droplet to boil. When boiling bubble formation encompassed the entire droplet, the temperature was assumed to be the boiling temperature. Incidentally, the rates from the boiling warming rate and the melting warming rate were similar, which makes sense as the temperature increased linearly due to the laser. This gives us confidence that both temperature indicators are satisfactory.

When considering CCR and CWR measurements, it is important to interpret the data with respect to the method with which it was gathered. For example, measurements made with DSC examine heat flow associated with crystallization and may give different results than convective methods that rely on the visual detection of ice, which itself is subjective. Even CCR and CWR data gathered by the same method, DSC, for example, may differ simply from the use of different sample sizes or pans of different roughness, which can alter nucleation in the sample. A separate problem arises in convective methods that rely on visual detection, where differences in lighting, image resolution, and experimenter subjectivity all can lead to different measurements of CCR and CWR. Figure S5 available in the Supplemental Materials shows that the CCRs of glycerol and PG exhibit the same trends, or have roughly the same slopes, with changing concentration as those reported in the literature [37–39]. DSC in general agreed better with our CCR data than convective cooling in capillary tubes; however, there was considerable variability in CCR data gathered via capillary tubes for glycerol than the data gathered here with freely exposed droplets. Additionally, extrapolations of CCRs of PG and glycerol to zero concentration give a CCR for pure water of the order of 10^7^ °C/min, consistent with a meta-analysis of CCR data in the literature [34]. The degree of offset between the measured values and those from the literature differ between PG and glycerol, which is to be expected, as the data from the literature were gathered via DSC for PG and via capillary quenching for glycerol. However, the exponential trend for CCR and concentration should be mostly independent of the characterization method, as was observed, with the magnitude of the offset between methods governed by the detection threshold of crystallization for vitrification. In both cases, since the offset appears to be constant with concentration and the exponential factor is roughly the same, the offset between methods can likely be attributed to differences in the thresholds for the detection of ice, where the cryotop quenching method for attaining CCRs likely has a lower threshold than DSC and capillary quenching. Ice detection in the capillary tube data was determined via x-ray diffraction, which was sensitive to ice fractions of roughly 1%, possibly higher than the detection threshold in our system. Also there was difficulty in measuring consistent cooling rates in the x-ray diffraction data set, since cooling rate was not linear due to differences between film and nucleate boiling. It is also possible that the cryotop induces more heterogeneous nucleation than the capillary tube and DSC, requiring larger CCRs to compensate for increased nucleation. No data obtained by convective methods go beyond 10^6^ °C/min. The ultimate limit of detection for convective warming has not been explored, but given the thermocouples currently available on the market, 10^6^ °C/min is likely approaching this limit (see further discussion in Supplemental Materials on the ASME Digital Collection).

**Fig. 5:**
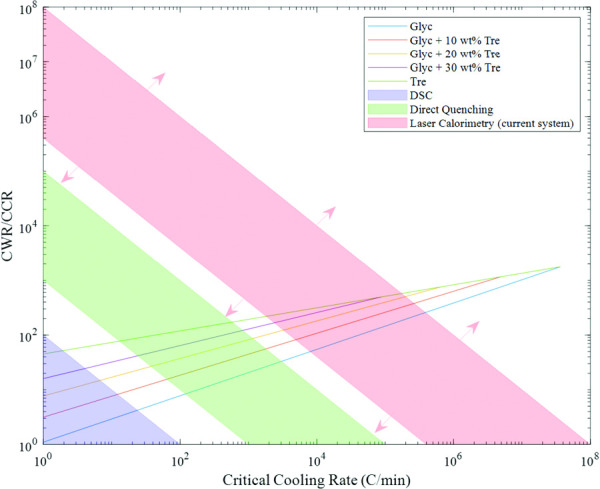
Effect of trehalose on the relationship between CWR/CCR and CCR of glycol-trehalose solutions. The CWRs and CCRs were determined from the two-species model based on the quenching and calorimetry experiments. The shaded regions show the different CWR measurement regimes corresponding to conventional DSC, direct quenching, and laser calorimetry with our current system. Different laser powers and high-speed cameras allow the boundaries of the laser calorimetry region to be extended.

There were larger uncertainties in the CWR experiments than in the CCR experiments, with generally better fitting data in the latter. This difference can be attributed to several causes. First, the droplets in the CCR experiments were at LN_2_ temperature when analyzed, which provided a static view of the degree to which the droplet was crystalized and allowed for the use of high-quality imaging to aid ice detection. In the CWR experiments, the high-speed video frames were less resolved than the images taken in the CCR experiments, making ice detection more difficult. Bubble formation from laser warming also made ice detection difficult in some cases by obscuring the ice underneath. The most prominent source of error was likely the temperature gradients within the warming droplet itself, with parts of the droplet warming faster than others. The source of these gradients and the implications for the CWR measurements are discussed in more detail below. It should be noted, though, that these temperature gradients also exist in convectively warmed and cooled droplets, so the relative magnitude of the gradients is important when considering error in the measurements. Future studies may incorporate more advanced image processing techniques to quantitatively determine the amount of ice in droplets systems, such as those used in the measurement of ice growth rates in thin films of CPAs [40].

### 4.2 Concentration Dependence of Critical Cooling Rate and Critical Warming Rate.

The CCRs measured for PG-trehalose and glycerol-trehalose solutions both showed a strong exponential dependence on concentration (Fig. 3). The CCR and CWR data for unary solutions of glycerol, PG, and trehalose, along with (Eq. (6)), allowed for calculations of the CWRs and CCRs of the mixtures of these solutions, which are shown as the model lines in Fig. 3. This calculation was contingent on the data in Fig. S8, available in the Supplemental Materials, which indicates the functional form of the concentration dependence of the exponential factor from (Eq. (4)). Figure S8, available in the Supplemental Materials on the ASME Digital Collection, shows how the exponential factor changes as the concentration of trehalose are altered, indicating that a linear relative concentration averaging of exponential factors is appropriate for a two-species solution, e.g., a constant change in concentration fraction leads to a constant change in the exponential factor. This result allows for a simple calculation of the changes in the CCR or CWR of a solution as the concentrations of its constituents are altered, provided that the exponential and pre-exponential factors for the individual constituents are known. Using this relation, one can identify better CPA mixtures by analyzing the effects of changes in the component concentrations on the CCR and CWR without requiring cumbersome and slow characterization. The exponential and pre-exponential factors for glycerol, PG, and trehalose are given in Table 2. We see some deviation from this model, but the general trends seem to be captured quite well. In Fig. 3(*c*), we see that the model-predicted lines converge toward a singular point near 50 wt % concentration of PG. Of course, in reality, this convergence does not occur and is simply the consequence of uncertainty being compounded with further extrapolation. In other words, the multispecies mixing model for CWR and CCR works best in the regime that the data were measured in barring some low-error measurement method that can measure across large sections of the concentration regime. Another option is to drastically lower the uncertainty in the concentration regime that the measurements were made in, thereby allowing for more confident extrapolations both forward and backward. The CCRs and CWRs for trehalose (Fig. 4) were measured for higher concentrations of 35–40 wt % while at lower concentrations they were then estimated by taking the exponential fit data for trehalose-glycerol and trehalose-PG mixtures in Fig. 3 and extending them to zero concentration for PG and glycerol. This data was used in combination with the measured values for trehalose to derive the exponential fit parameters for trehalose, which are shown in Table 2 along with those for PG and glycerol. The low-concentration extensions for both the CCRs and CWRs of trehalose line up with the measured data, implying that the estimation of the CCRs and CWRs for lower concentrations of trehalose is accurate, assuming that the exponential dependence on concentration behaves similarly at low concentrations as it does at moderate concentrations. Additionally, it was shown that trehalose has a larger effect on the CCR and CWR of glycerol than on those of PG, though the exact mechanism causing this is unclear. One proposed mechanism is that added sugars affect the CPA glass transition temperature, thereby altering the vitrification properties [34,41]. It is also possible that the large difference in viscosity between PG and glycerol plays a role. We offer an additional explanation based on (Eq. (6)). If we compare two solutions of equal weight percent trehalose and glycerol/PG, we see that the only differing parameter is $\alpha_{j}$. Since we have already determined that PG-water solutions have a higher $\alpha_{j}$ that glycerol-water solutions, it follows directly that the addition of trehalose, or for that matter, any other CPA, will affect the CCR and CWR of the glycerol solutions more than those of the PG solutions with respect to concentration.

**Table 2:** Exponential and pre-exponential factors for the cooling and warming rate dependence of the CCR and CWR, respectively

	*A* Pre-exponential factor CCR (°C/min)	*A* Pre-exponential factor CWR (°C/min)	α Exponential factor CCR (wt %^−1^)	α Exponential factor CWR (wt %^−1^)
Trehalose	3.64 × 10^7^	6.42 × 10^10^	0.207	0.243
PG	5.22 × 10^7^	7.26 × 10^10^	0.325	0.368
Glycerol	4.96 × 10^7^	2.18 × 10^11^	0.216	0.295

The CCRs and CWRs of CPAs are known to depend exponentially on concentration in several different concentration regimes [33,34,38]. Unfortunately, due to the constraints on the methods for measuring the CCR and CWR, one method cannot be used over the entire concentration regime, as shown in Fig. 5. That is to, say, it is not that data collected by different methods cannot be directly compared but that some discrepancies are to be expected when comparing CCRs and CWRs from different methods. Thus, we cannot be certain that the exponential relationship applies across the entire concentration regime or if the limitations of the methods themselves mask the true relation. As a result of the method constraints, measurements are made by DSC in the low-rate regime (<100 °C/min), by convective cooling/warming via quenching in the moderate-rate regime (10^3^–10^6^ °C/min), and by laser calorimetry for CWR measurements only in the high-rate regime (>10^6^ °C/min). As of this date, there exists no method capable of volumetric cooling in this high-rate regime. Each method has unique limitations. DSC is limited by the ability to stably extract or add heat to a sample and by the sensor sensitivity. Convective methods rely on thermocouples, which inherently have a maximum detectable rate. The thermocouple also adds thermal mass and possible nucleation sites to a system, thereby altering the CCR or CWR measurement. The key downside of convective methods is that outside of the lumped regime (i.e., larger droplets) temperature gradients can be appreciable so that measured temperatures at one point are not representative of the whole system. Additionally, because the way to attain larger warming rates is to decrease the sample volume, different warming rates necessarily have different cooling rates prior to warming in convectively warmed systems.

The general question now arises as to how the CWR should be measured, for instance, whether all samples should be cooled at the same rate before warming or whether samples should be cooled as fast as possible before warming. At higher CPA concentrations, which correspond to lower warming rates, this requirement does not pose much of a problem, as cooling rates much larger than the CCR are easily attainable, but this is not true as the CPA concentration is decreased. Laser calorimetry is still limited by size on cooling, but relatively uniform warming at arbitrary rates is achievable, allowing for a much more robust characterization of the CWR along with the ability to attain much larger warming rates. As the CPA concentration decreases and the CCR increases, temperature uniformity on warming becomes much more important, as conduction no longer smooths out the temperature gradients on the shorter timescales during warming. Future experiments involving less powerful lasers may make it possible to determine CWRs from 100 °C/min up to 10^8^ °C/min, all being warmed uniformly with the cooling rate under precise control, thereby eliminating much of the error between characterization methods. It should also be noted that coupling high-power laser calorimetry with faster droplet cooling approaches will make possible the characterization of CWRs of CPAs in the 1–2 M regime (∼10–20 wt %), with rates exceeding 10^8^ °C/min.

In the literature, extrapolations of CWR data from moderate-concentration CPAs out to zero concentration yield extreme differences in the CWR of pure water, though the measured data follow exponential fits quite well in the regimes they were measured in Refs. [27] and [34]. The estimated values of the CCR and CWR for water that are derived from the PG, glycerol, and trehalose data are shown in Table 3. The same treatment of the literature data for the CCR from moderate-concentration CPAs also yielded differences in the CCR of water but to a much lesser extent. These differences in the extrapolated CWRs and CCRs of water could be caused by errors in the measurement methods themselves or by the possibility that the CCR and CWR do not follow an exponential dependence on concentration throughout the entire concentration regime. The discrepancies between the extrapolated values from different CPAs are much more apparent in the CWR measurements than in the CCR measurements, possibly implying the presence of systemic errors in the measurement methods themselves. There is also evidence that the exponential relationship between CWR/CCR and concentration does not apply to the entire concentration regime, as it is known to break down at high concentrations; however, it is unclear whether this breakdown is due to the different characterization methods applied in the different regimes shown in Fig. 5 or differences in sample volume or whether it is a fundamental property of CPAs [19,27].

**Table 3:** Estimated values of the CCR and CWR of pure water from the experimental values attained from LN_2_ quenching and laser calorimetry of PG, glycerol, and trehalose solutions

	Experimental
CCR	3.64–5.22 × 10^7^ (°C/min)
CWR	0.64 – 2.18 × 10^11^ (°C/min)

Using the rotational correlation time for water, the time it takes for a water molecule to rotate one radian via diffusion, we can estimate an upper bound on the CWR for pure water. This correlation time has been measured at 1.7 ps [42], which means that if water is warmed from its glass transition temperature to the melting temperature faster than 1.6 × 10^15^ °C/min, the water molecules will not have enough time to rotate and align in the new crystal formation, assuming a maximum necessary rotation of 180 deg. This of course is an overestimate, as the rotational correlation time for supercooled and vitrified water is certainly larger than that of room-temperature liquid water. Therefore, we can infer that extensions of CWRs beyond 10^15^ °C/min are erroneous and that either the exponential model breaks down or there is an error in the measurement.

The ratio of CWR to CCR was also shown to be exponentially dependent on concentration based on the multispecies model developed and data gathered throughout the experiments (see Figs. 5 and S12 available in the Supplemental Materials on the ASME Digital Collection). This dependence is to be expected, as both the CCR and CWR are exponentially dependent on concentration. It was found that the lower the concentration is, the larger the difference between the CWR and CCR, with estimates of the difference for pure water being a factor of roughly 3000–3500. This result has unfortunate implications for cryopreservation. As the concentrations of CPAs are lowered to avoid CPA toxicity, not only does the CCR rise, but the CWR rises at an even faster rate, meaning that extremely fast warming rates of the order of 10^9^ °C/min are required to rewarm CPA concentrations under 15 wt %, at least for the CPAs studied. Unless CPAs that have a much lower CWR/CCR ratio exist, it seems unlikely that the current methods will successfully rewarm samples with CPA concentrations much under 15 wt % without appreciable ice formation. Figure 5 illustrates this difference with a plot of CWR/CCR versus CCR. One can see that in a two-species solution of glycerol and trehalose, the CWR/CCR curves of the individual species form upper and lower bounds to the CWR/CCR curves of mixtures of the two species. This feature arises because the exponential factors of a unary trehalose solution and a unary glycerol solution are almost identical, and so the exponential factor of glycerol-trehalose mixtures has almost no concentration dependence. In contrast, for PG-trehalose, shown in Fig. S12 available in the Supplemental Materials, these lines cross, which can be attributed to the concentration dependence of the exponential factor between PG and trehalose. Since the minimum warming rate of the laser was 400,000 °C/min, a line taken from 400,000 °C/min on the *y*-axis to 400,000 °C/min on the *x*-axis in Fig. 5 arrives at the lower bound of the red-shaded laser calorimetry region. This red-shaded region could be broadened with the implementation of lasers of higher or lower power as well as faster high-speed cameras. The volume of the sample is also a limiting factor in laser calorimetry. In principle, much smaller droplets than were used in the study could be constructed and measured via laser calorimetry; however, this measurement would require an additional focusing lens for the high-speed camera. Additionally, it should be noted that this upper limit on the warming rate measurement was not reached in this study and that vitrification on cooling was actually the limiting factor.

### 4.3 Warming Rate Measurement.

The high-speed camera had the ability to pick up near-infrared radiation, which was advantageous for synchronizing the camera with the laser pulse, allowing for accurate estimation of the start of the laser pulse. By using this time point with temperature indicators at the melting point and boiling point of the CPA, we could predict the warming rate in the droplet during laser warming. This treatment is contingent on an absence of substantial superheating of the liquid and solid phases in the droplet. If this superheating were to occur, we would expect major differences in the warming rates calculated from melting and boiling. Fortunately, there was not a significant difference between these two metrics, at least with respect to the error in the system. That is, the interdroplet variability outweighed any differences between warming rates calculated with these two metrics.

The two most substantial sources of error in the laser calorimetry experiments are thought to be the droplet shape and random scattering due to bubble and crystal formation, both of which affect the absorption profile in the droplet and induce thermal gradients. In some cases, a ring of ice formed around the base of the droplet at the beginning of a set of experiments. This ice formation was due to the curvature of the droplet being too large and causing lensing of the laser, as seen in Fig. S10 available in the Supplemental Materials on the ASME Digital Collection. Over the course of a set of experiments, residue from the droplets would build up on the cryotop and increase the effective hydrophilicity of the cryotop surface, leading to flatter droplets and a reduction in the number of droplets with this ring feature. Warming rates taken from droplets with an apparent ice ring at the base were simply ignored. The droplet shape also changed during warming, which altered the absorption profile in the droplet by changing how the droplet bent the incoming laser over the duration of the pulse. Due to the random nature of this phenomenon, it is difficult to quantify its overall effect on the warming rate measurement variability. Bubble and crystal formation in the droplet during warming also induces laser scattering, but the extent to which it affects the measurement variability and thermal gradients is also unknown and would make an interesting topic for future study.

**Fig. 6:**
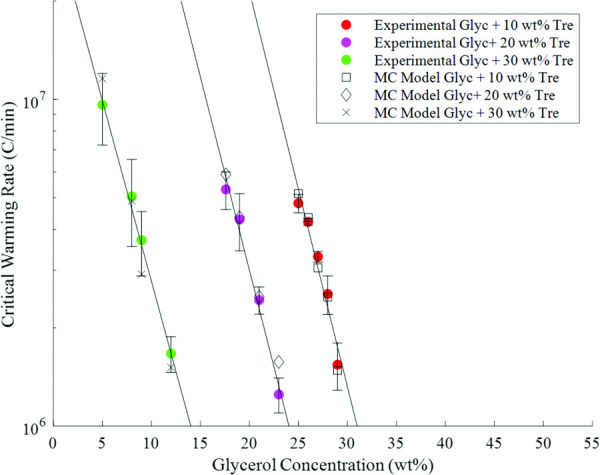
Comparison of the CWRs measured via laser calorimetry and the warming rates calculated from the MC model based on the laser settings and gold concentrations in the CWR experiments

The MC model also provided an independent metric for the warming rate calculations in the laser calorimetry experiments. Figure 6 shows the measured warming rates and the predicted warming rates calculated based on the absorption properties of the GNR solution, shown in Fig. S9 available in the Supplemental Materials, and the laser energy settings used on samples warmed at the CWR. Most of the model-predicted rates are within the error bars of the CWR data gathered via laser calorimetry. This agreement in the data leads us to believe that the warming rates measured in the laser calorimetry experiments are reasonable approximations of the true values since two independent methods gave similar results. The MC model was also able to accurately predict the ice ring that forms along the outer edge of the droplet in cases of high droplet curvature. The success of the MC model in accurately predicting the warming rates and temperature gradients in the droplets suggests that it is a powerful tool for studying warming in warming regimes not yet attainable with current technology (>10^8^ °C/min) as well as at scales that are invisible to the current high-speed camera, such as on the cellular level.

## 5 Conclusion

The CWRs ranging from 0.4 × 10^5^ to 10^7^ of glycerol-trehalose and PG-trehalose solutions were measured via laser calorimetry, the fastest such rates measured to date. The CCRs of these same CPAs were also measured via direct quenching into LN_2_. The data for CCR and CWR both were consistent with extrapolations from literature data measured at higher concentrations. Additionally, by measuring the CWR and CCR dependence of PG and glycerol for varying concentrations of trehalose, a model describing CCRs and CWRs for CPA mixtures was developed and verified, allowing for calculations of the CCR and CWR of CPA mixtures given only their constituent solutions' individual CCRs or CWRs through the use of (Eq. (6)). This model allows for the analysis of CPA cocktails without the need to make and characterize them, making CCR and CWR optimization much more efficient. The data as well as model parameters for fitting the data for PG, glycerol, and trehalose were provided. We now provide a framework, through laser calorimetry, for measuring the CWRs of CPAs in not only the low-concentration regime but throughout the concentration regime by simply altering the laser power, eliminating much of the uncertainty that arises when comparing CWRs measured by different techniques at different size and rate scales. Together, this work reported new experimental and theoretical results linking the CWR and CCR for a given CPA within an otherwise inaccessible low-concentration measurement regime (20–40 wt %).

## Funding Data

- NIH/NHLBI (Grant No. R01HL135046-01; Funder ID: 10.13039/100000050).
- NIH/NHLBI (National Heart, Lung, and Blood Institute) (Grant No. R44MH122118; Funder ID: 10.13039/100000050).
- NIH/NIDDK (National Institute of Diabetes and Digestive and Kidney Diseases) (Grant No. R01DK117425-01; Funder ID: 10.13039/100000062).
- Minnesota Sea Grant, University of Minnesota (Funder ID: 10.13039/100005774).
- NSF ERC for Advanced Technologies for the Preservation of Biological Systems (ATP-Bio) (Funder ID: 10.13039/100000001).

## Author's Contributions

JK, HN, KK, and JB conceived of the concept. JK, KK, and JB designed the experiments. JK, LZ, and YL performed the experiments. JK and YL analyzed the data. All authors contributed to the writing of the manuscript.

## Supplementary Material

Supplementary Material Supplementary PDF
