# Supplementary material for: Ultra-Rapid Laser Calorimetry for the Assessment of Crystallization in Low-Concentration Cryoprotectants
Source: J Heat Transfer. 2022 Feb 7;144(3):031207. doi: 10.1115/1.4052568 (PMC8823201; doi:10.1115/1.4052568)
Supplement: Supplementary Material — Supplementary PDF [file Supplementary_Material_HT-21-1359.zip › Supplementary_Material_HT-21-1359.pdf]

CWR and CCR for a given CPA within an otherwise inaccessible low-concentration measurement regime (20-40 wt%).

SUPPLEMENTAL MATERIAL

S1. CCR measurements

Two CPA mixtures were considered for this study: a PG-trehalose-water solution and a glycerol-trehalose-water solution, and the concentration fractions of the individual CPA chemicals were varied. A cryotop consisting of an 80-µm-thick strip of polypropylene adhered to a 5-mm-diameter wooden rod was constructed to hold the sample.[43,44] Next, a 25-µm-diameter Type T unsheathed fine-gauge thermocouple was fastened to the cryotop with cyanoacrylate so that the junction of the thermocouple resided near the leading edge of the cryotop. For data acquisition, a StingRay DS1M12 from EasySync Ltd connected the thermocouple directly to a laptop. This method of thermocouple data acquisition with a cryotop builds directly off of methods outlined in Kleinhans 2010.[45] The thermocouple-cryotop assembly was then attached to a mechanical jig that controlled its height. This design feature allowed for the controlled plunging of the sample into liquid nitrogen (LN_2), thereby increasing the repeatability between trials, shown in *FIG. S1*. A micropipette was used to place a droplet of the CPA solution on the cryotop so that the thermocouple junction was directly in the center of the hemispherical droplet. Then, the droplet was plunged into LN_2, allowed to reach thermal equilibrium with the LN_2, and then raised up to assess ice formation by visual assay. Samples with any traces of ice were classified as not vitrified. *FIG. S2* provides a demonstration of the vitrification criterion used. The temperature data gathered were then labeled as either vitrified or not vitrified.

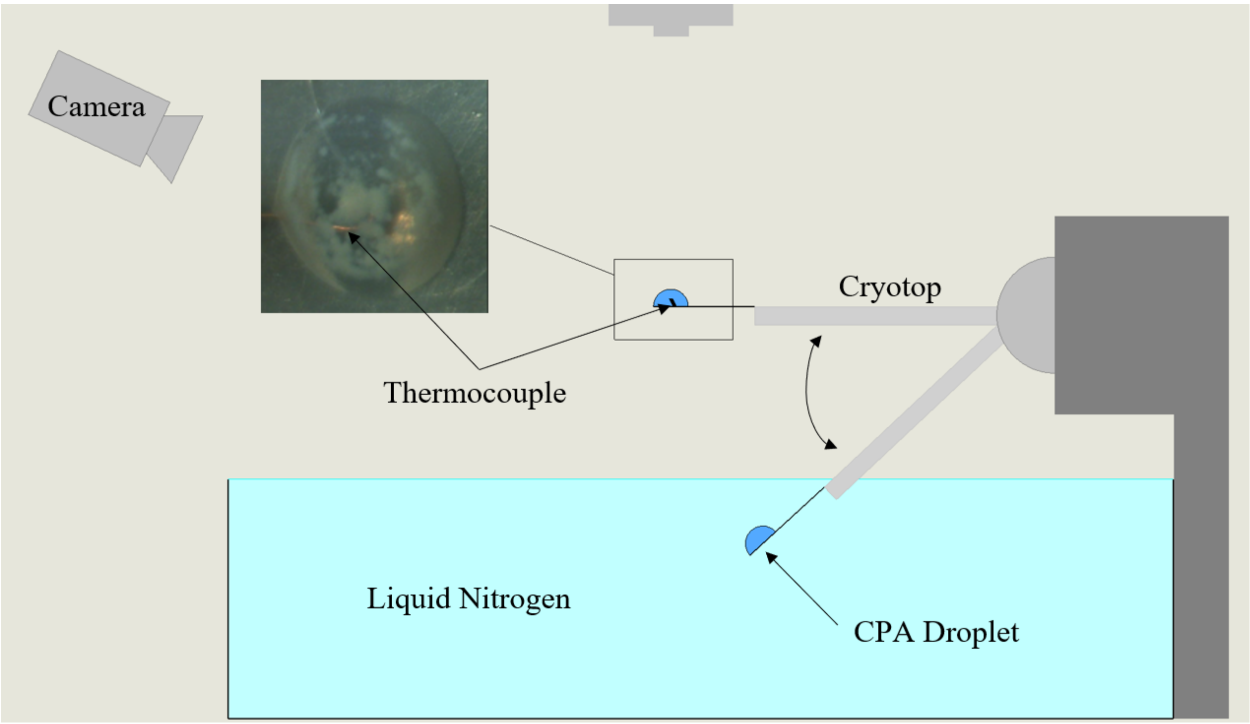

FIG. S1. Schematic of CCR experimental setup

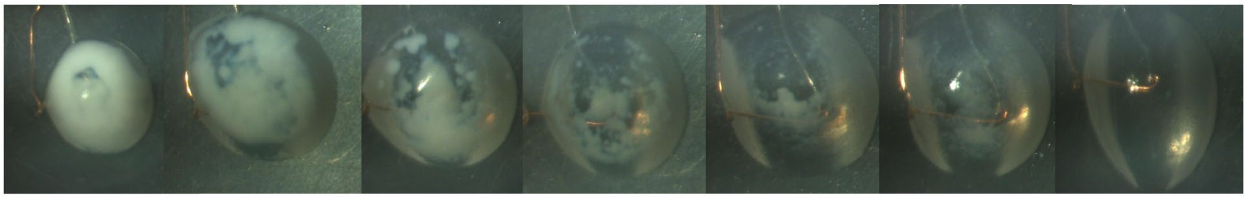

FIG. S2. Visual assay to assess ice formation. Images show a gradually increasing concentration of PG from left to right and its effect on the amount of ice detected. The right-most image is the only image we classified as vitrified.

Temperature data were then exported to MATLAB for analysis. A best fit line of temperatures between -20 °C and -190 °C was used to determine the cooling rate in the droplet, as shown in *FIG. S3*. The temperature profiles were approximately linear in this range of temperatures for droplets smaller than 10 µL. The cooling rate was controlled in this experiment through volume modulation of the droplet, shown in *FIG. S4*. There are some differences between the measured data in *FIG. S4* and the data gathered in this study. Reference 34 and 35 used DSC to extract

kinetic parameters to estimate the CCRs using Boutron's model P. Boutron, Cryobiology 23, 88–102 (1986). Reference 36 uses x ray diffraction of quartz capillary tubes which show high variability in CCR. Their data also follows roughly the same trend as ours but is offset. This may be due to several factors. First, they used XRD to validate vitrification, which may be less sensitive than visual detection, making their measurements lower than ours. Additionally, they had difficulty measuring cooling rate as it was not linear for many of their samples, due to film vs nucleate boiling during cooling in liquid propane and liquid nitrogen, likely causing the large uncertainty in measurement.

To determine the CCRs of the CPAs, the following technique was employed. First, a 5-μL droplet of CPA was plunged into LN_2, and its cooling rate was recorded. Droplets larger than 5 μL gave inconsistent cooling rates due to cracking, so the starting volume for the CCR experiments was limited to 5 μL. If this solution vitrified, the CPA concentration was too high for the CCR to be measured by this particular method, and the CPA concentration was simply lowered until the 5 μL droplet did not vitrify. The droplet volume was then decreased until no ice could be visually detected after plunging the droplet in LN_2. The cooling rate measured at this point was called the CCR. Using this method, CCRs between 5,000 and 100,000 °C/min were attainable.

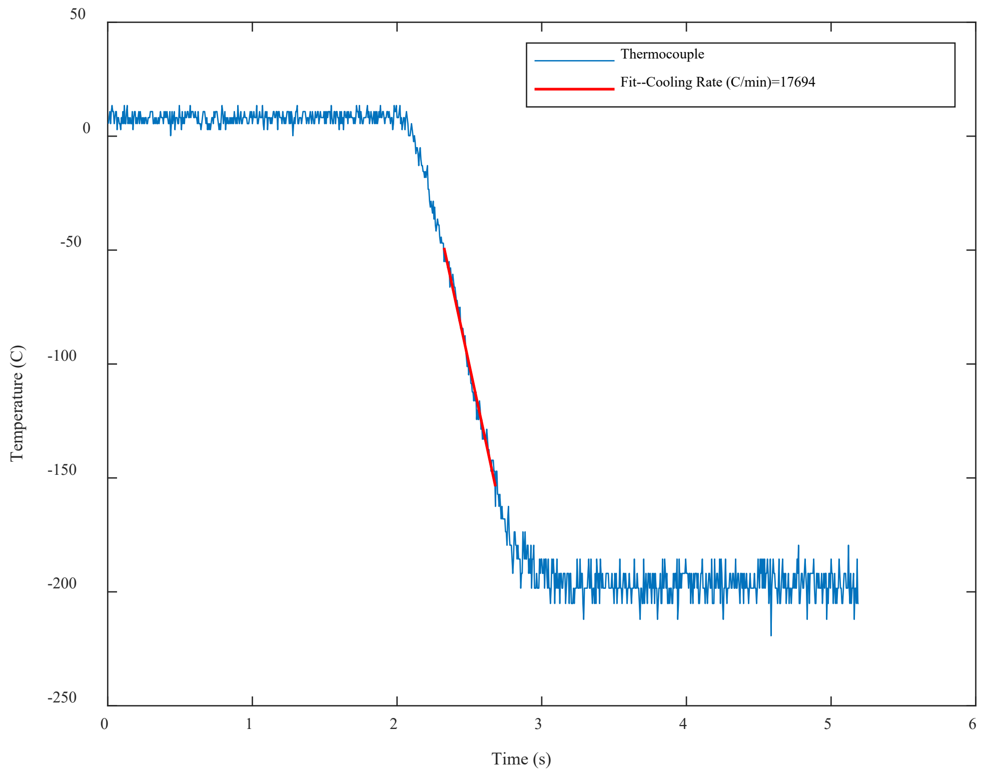

FIG. S3. Thermocouple data obtained from LN_2 quenching of a 0.9-µL droplet of PG.

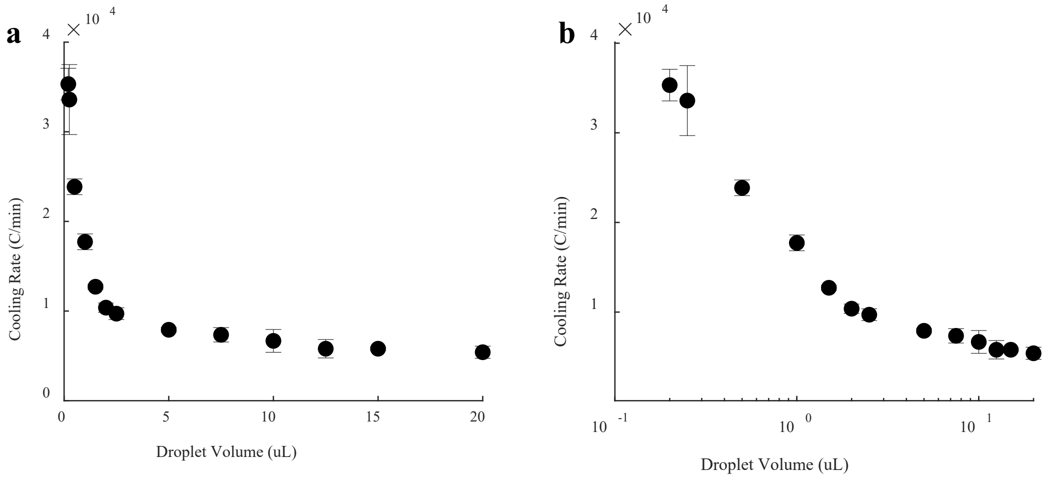

FIG. S4. Dependence of the cooling rate on droplet volume for cryotop quenching into LN_2, (a) linear and (b) log-scale concentration.

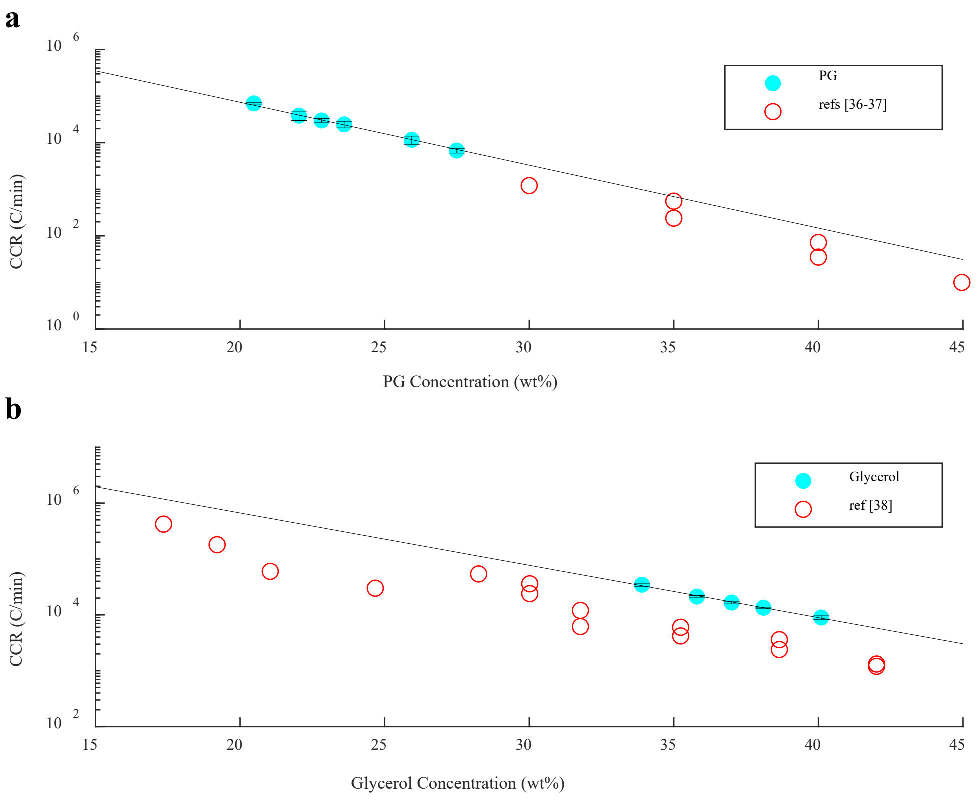

FIG. S5. CCRs attained via measurement by thermocouple and LN_2 quenching compared with the critical cooling data available in the literature for PG (a) and glycerol (b). Error bars represent the difference between the lowest measured cooling rate that achieved vitrification and the largest measured cooling rate that did not achieve vitrification.[36–38]

S2. Mixture model

We come across the relation for a mixture of two CPAs by this simple method. We know that at $c_1 = 0$ the $R_{1,2} = R_2$ since it is no longer a mixture but a unary solution, likewise for $c_2 = 0$. Then the following function maintains both these constraints.

$$R_{1,2} = Ae^{-\alpha_1 c_1}e^{-\alpha_2 c_2}e^{-f(\alpha_1,\alpha_2,c_1,c_2)c_1 c_2} \tag{6}$$

We notice in *FIG. S6* that this formula for mixing comes naturally from the graphical representation of how the mixture of two CPAs should affect CCR or CWR, so that $R_{1,2}$ contains

both R_1 and R_2, and that the mixing terms is some function of the individual components, all while maintaining exponential dependence on concentration.

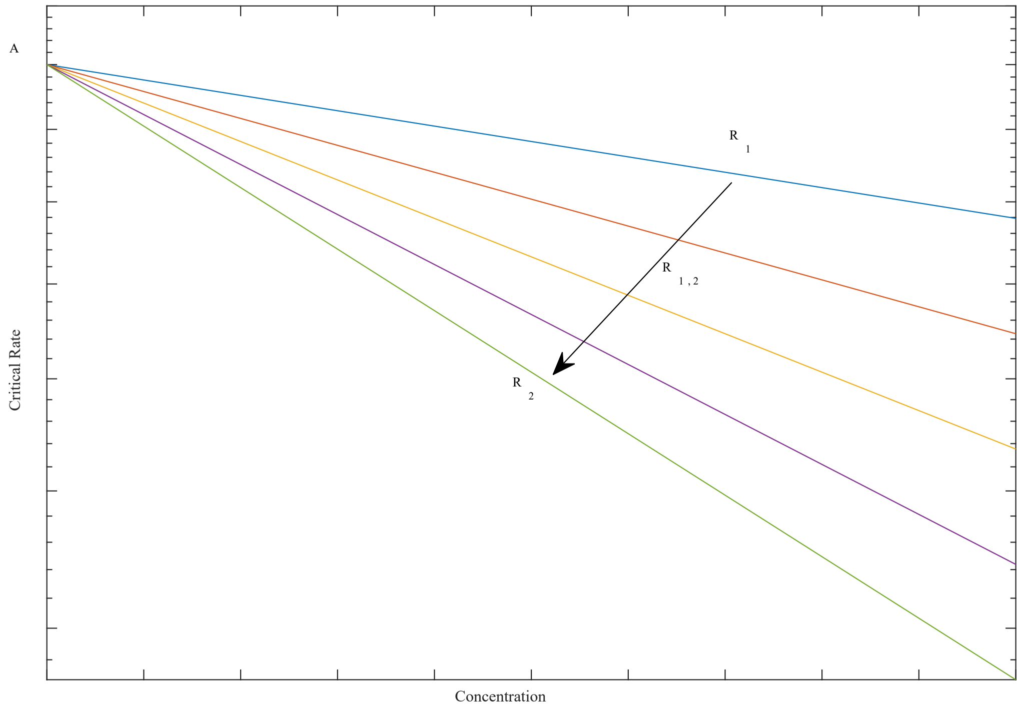

FIG. S6. Shows how the CCR or CWR is changed when two CPAs are mixed together (assuming equal pre-exponential factors), where concentration simply refers to the total concentration of all components of the CPA.

In theory, the preexponential factor should be the same for all cryoprotectants, but in actuality, there will be differences due to error and uncertainty, and breakdowns in the exponential trend. To combat this variation, the pre-exponential factor can be considered to be the average of those for each individual species i in the mixture.

$$A_{avg} = \frac{1}{N}\sum_{i=1}^{N} A_i \tag{7}$$

So, (Eqn. 6) for $t$hen becomes

$$R_n(c_1, \dots, c_n) = A_{avg} \prod_{i=1}^{n} e^{(-\alpha_i c_i)} \prod_{1 \le i < j \le n} e^{-\frac{\alpha_j - \alpha_i}{\chi_i} c_i c_j} \tag{8}$$

For CPA mixtures with constituents that have pre-exponential factors that vary considerably, a similar treatment must be applied to the pre exponential factor as was done in (Eqn. 4). For a mixture of two species if the linear concentration average that applied for the exponential factor also applies to the pre-exponential factor, we arrive at (Eqn. 9).

$$A_{mix} = A_1 + \frac{A_2 - A_1}{\chi_2} c_2 \tag{9}$$

At this moment it is not known if (Eqn. 9) is valid. Verification is left to a future study, though we hypothesize this behavior which is seen in *FIG. S7.*

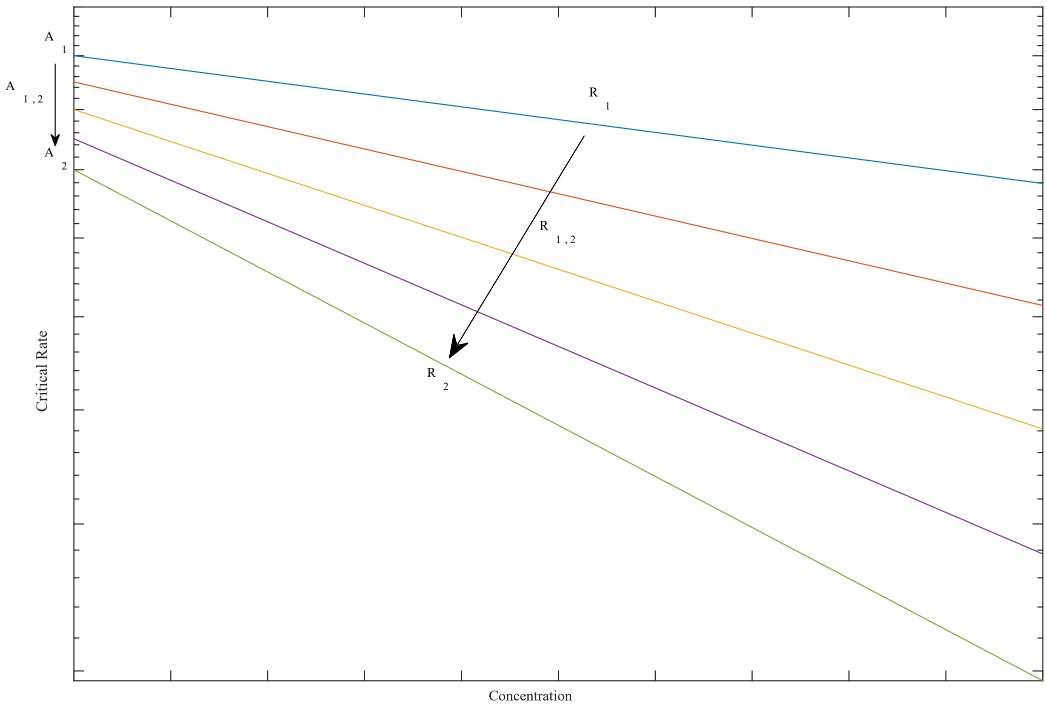

FIG. S7. Shows how the CCR or CWR is changed when two CPAs are mixed together (assuming mixing of pre-exponential factors), where concentration simply refers to the total concentration of all components of the CPA.

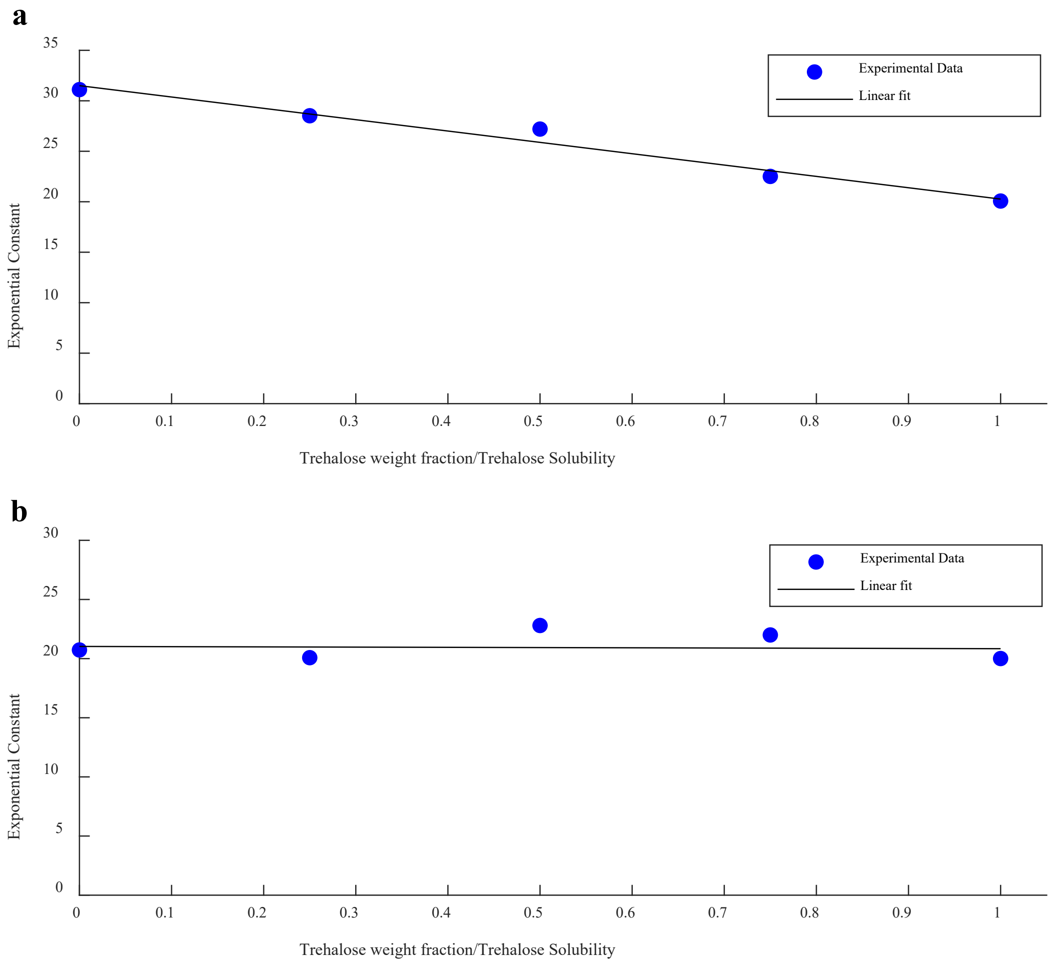

FIG. S8**.** Concentration dependence of the exponential factor for a PG-trehalose mixture (a) and a glycerol-trehalose mixture (b).

S3. Numerical modeling of laser warming

The validity of the CWR measurements made via laser calorimetry is contingent on the uniformity of the temperature profile during warming. If large gradients exist throughout the droplet, the calculated warming rate may not be accurate. Temperature uniformity during the plasmonic heating of GNPs has been studied via MC modeling previously in Liu et al. 2020.[35,46] That MC model of laser warming was adapted to the laser calorimetry setup used in this study, which incorporated the scattering, absorption, reflection, and refraction interactions of

laser photons with droplets. First, a photon path Γ through the system consisting of linear segments of length s_i was constructed, where s_i is a function of the absorption coefficient μ_a, scattering coefficient μ_s, and a uniform random variable $U_{[0,1]}$ between 0 and 1.

$$s_i = -\frac{\ln(U_{[0,1]})}{(\mu_a + \mu_s)} \tag{10}$$

Each of these segments can be thought of as a photon path prior to a scattering event. The polar angle θ and azimuth angle φ between subsequent segments s_i and s_{i+1} are governed by (11) and (12), respectively, where g is the scattering anisotropy coefficient.

$$\cos(\theta) = \frac{\left(1 + g^2 - \left(\frac{1 - g^2}{1 - g + 2gU_{[0,1]}}\right)^2\right)}{2g} \tag{11}$$

$$\varphi = 2\pi U_{[0,1]} \tag{12}$$

Photons that cross the droplet boundary are reflected via Snell's law with respect to the normal direction at the point of intersection. Upon reflection, photon energy is diminished according to the Fresnel equations. By applying the expected value to Beer's law along Γ , we note that the photon energy before and after photon scattering is given by (13), while the energy absorbed is given by (14).

$$E_{i+1} = \frac{\mu_s}{\mu_a + \mu_s} E_i \tag{13}$$

$$E_{absorbed} = \frac{\mu_a}{\mu_a + \mu_s} E_i \tag{14}$$

After a sufficient number of scatterings, absorptions, and reflections to cause the photon energy to fall below some small threshold, the photon trajectory will be terminated. After simulating millions of photons in the environment, we can generate an accurate approximation of the heat source

function or specific absorption rate (SAR). Once the SAR is calculated, it can then be used as input into a COMSOL model of heat transfer within the droplet to determine the spatial-temporal temperature profile via finite element analysis.

$$\rho c_p \frac{\partial T}{\partial t} - \nabla \cdot (k \nabla T) = SAR \tag{15}$$

$$T(\boldsymbol{x}, 0) = T_{LN2}, \quad \boldsymbol{x} \in \Omega \tag{16}$$

$$-k \nabla T(\boldsymbol{x}, 0) = h\big(T_a - T(\boldsymbol{x}, 0)\big), \quad \boldsymbol{x} \in \partial\Omega \tag{17}$$

Where Ω consisted of a hemisphere with diameter 1.24 mm, corresponding to a .5 µL droplet, T_{LN2} was liquid nitrogen temperature 77K, T_a was ambient temperature 273K, and the heat transfer coefficient was taken to be 100 $Wm^{-2}K^{-1}$.[47]

To verify that there is not extreme local heating around each nanoparticle, causing a breakdown of the global heating assumptions, we reference the solution to the transient heat equation around a nanoparticle.[48]

$$T(r,t) - T_\infty = \frac{dQ_{nano}}{4\pi r k \Delta t}[\text{erfc}\left(\frac{r - r_p}{2\sqrt{\alpha t}}\right) - \exp\left(\frac{r - r_p}{r_p} + \frac{\alpha t}{r_p^2}\right) \text{erfc}\left(\frac{r - r_p}{2\sqrt{\alpha t}} + \frac{\sqrt{\alpha t}}{r_p}\right) \tag{18}$$

Where dQ_{nano} is the power generated by a single nanoparticle, r_p is the nanoparticle diameter, Δt is the pulse duration of the laser, k is the thermal conductivity, and α is the thermal diffusivity. We note that at the nanoparticle surface, the steady state temperature difference between the nanoparticle and its surroundings is:

$$\Delta T_{nano} = T(r,t) - T_\infty = \frac{dQ_{nano}}{4\pi r k \Delta t} \tag{19}$$

For the pulse energy of 60 joules, pulse duration of 1 ms, nanoparticle density 5×10^{16} np/m^3, and droplet volume of .5 µL, we estimate the ΔT_{nano} = .006 K. Thus, we can be confident that localized heating around the nanoparticles is inconsequential and that the global heating approximation is valid. Additionally, an alternate rational for validating bulk heating in laser warmed nanoparticles is given by analyzing thermal confinement.[49]

For each critical warming measurement, the MC model was used to predict the warming rate of the droplet by using the optical properties of the GNR concentration used in that droplet as well as the laser settings at the determined CWR. The thermal properties of PBS+2M glycerol were used in the finite element modeling for all CPA concentrations modeled, as this mixture provided the closest temperature-dependent data to the CPAs used in this study.[50] To adequately describe laser warming, the optical properties of the GNPs need to be measured. A linear relation between the absorption coefficient and concentration of the GNR solution was determined via UV-VIS spectroscopy, as shown in *FIG. S9*. The scattering coefficient and scattering anisotropy coefficient were estimated via the relation found in Liu et al. 2020. The droplet in the COMSOL modeling had a contact angle of 90 degrees, making it a perfect hemisphere. In reality, the droplet spread out more, eliminating the large degree of lensing that occurred at the edges. Regardless, the warming rates from the COMSOL model of the perfect hemisphere were essentially identical to those with a slightly flatter shape. Due to this lack of difference, the hemispherical droplet shape was used in the modeling for ease of defining the boundary.

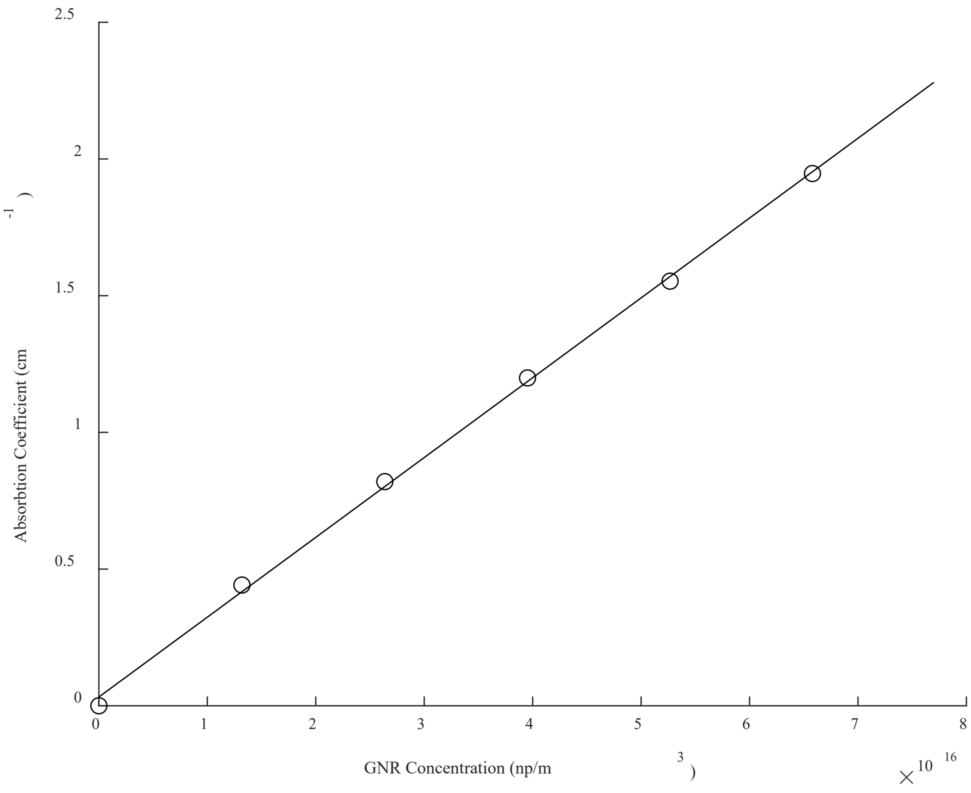

FIG. S9. Absorption coefficient vs concentration of GNR solution measured via UV-VIS spectroscopy.

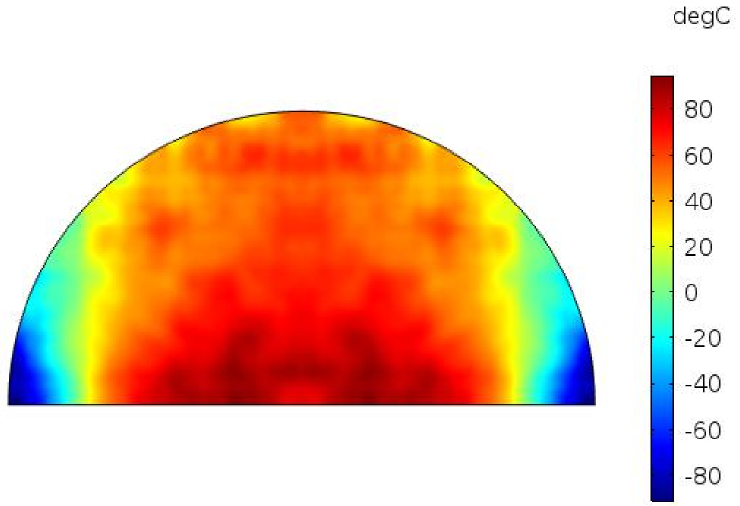

FIG. S10. Temperature profile in a droplet of GNR solution right at the end of the laser pulse.

S4. Warming Rate Measurements

VIDEO LINKS IN CAPTION

FIG. S11. Videos of laser warming for a droplet warmed below the critical warming rate (a) https://www.youtube.com/watch?v=TY5WjSFbjsw and above the critical warming rate (b) https://www.youtube.com/watch?v=q4aM0Ekpiq8

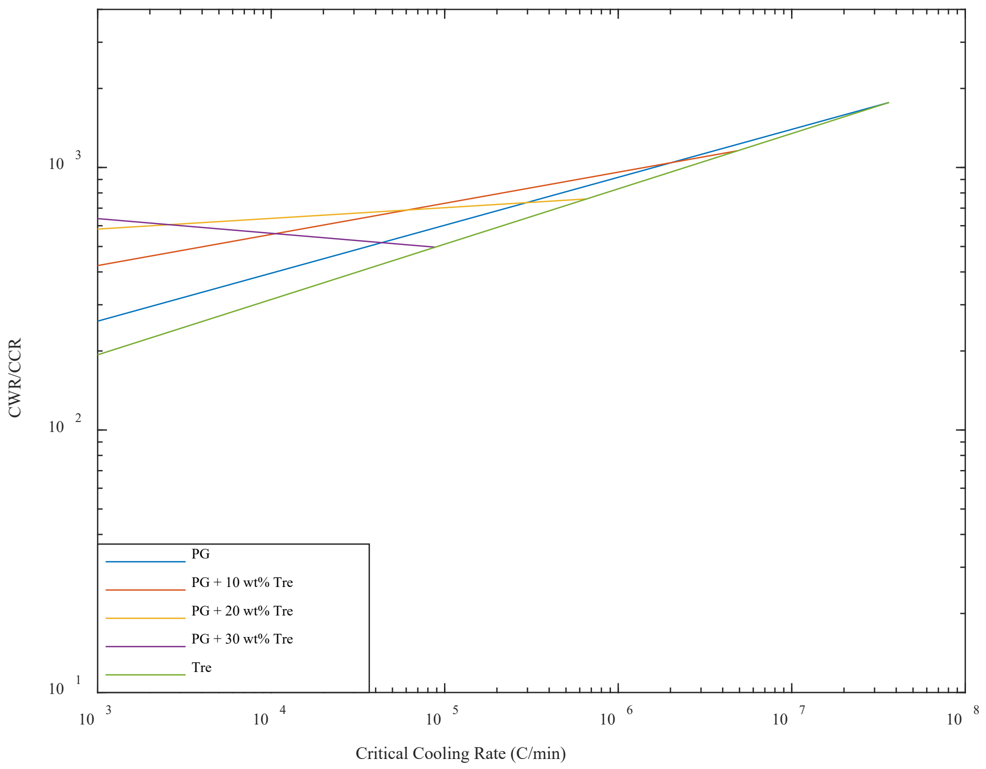

FIG. S12. Effect of trehalose on the relationship between CWR/CCR and CCR of PG-trehalose solutions. The CWRs and CCRs were determined from the two-species model based on the quenching and calorimetry experiments.

AUTHOR'S CONTRIBUTIONS

JK, HN, KK, and JB conceived of the concept. JK, KK, and JB designed the experiments. JK, LZ, and YL preformed the experiments. JK and YL analyzed the data. All authors contributed to the writing of the manuscript.

ACKNOWLEDGEMENTS

We would like to acknowledge the following funding sources.

NIH/NHLBI R01HL135046-01

NIH/NHLBI R44MH122118

NIH/NIDDK R01DK117425-01

Minnesota Sea Grant

NSF ERC for Advanced Technologies for the Preservation of Biological Systems (ATP-Bio)

REFERENCES

[1] MAZUR, P., 1988, "Stopping Biological Time: The Freezing of Living Cells," *Ann. N. Y. Acad. Sci.*, **541**(1), pp. 514–531.

[2] Wolkers, W. F., and Oldenhif, H., 2015, *Cryopreservation and Freeze-Drying Protocols*, Springer-Verlag, NY.

[3] Wowk, B., 2007, "How Cryoprotectants Work," *Cryonics*, **3**, pp. 28.

[4] Elliott, G. D., Wang, S., and Fuller, B. J., 2017, "Cryoprotectants: A Review of the Actions and Applications of Cryoprotective Solutes That Modulate Cell Recovery from Ultra-Low Temperatures," *Cryobiology*, **76**, pp. 74–91.

[5] Muldrew, K., and McGann, L. E., 1990, "Mechanisms of Intracellular Ice Formation," *Biophys. J.*, **57**(3), pp. 525–532.

[6] Takamatsu, H., and Zawlodzka, S., 2006, "Contribution of Extracellular Ice Formation and the Solution Effects to the Freezing Injury of PC-3 Cells Suspended in NaCl Solutions," *Cryobiology*, **53**(1), pp. 1–11.

[7] Best, B. P., 2015, "Cryoprotectant Toxicity: Facts, Issues, and Questions," *Rejuvenation Res.*, **18**(5), pp.

422–436.

[8] Fahy, G. M., Lilley, T. H., Linsdell, H., Douglas, M. S. J., and Meryman, H. T., 1990, “Cryoprotectant Toxicity and Cryoprotectant Toxicity Reduction: In Search of Molecular Mechanisms,” *Cryobiology*, **27**(3), pp. 247–268.

[9] Khosla, K., Wang, Y., Hagedorn, M., Qin, Z., and Bischof, J., 2017, “Gold Nanorod Induced Warming of Embryos from the Cryogenic State Enhances Viability,” *ACS Nano*, **11**(8), pp. 7869–7878.

[10] Guthrie, H. D., Liu, J., and Critser, J. K., 2002, “Osmotic Tolerance Limits and Effects of Cryoprotectants on Motility of Bovine Spermatozoa,” *Biol. Reprod.*, **67**(6), pp. 1811–1816.

[11] Szurek, E. A., and Eroglu, A., 2011, “Comparison and Avoidance of Toxicity of Penetrating Cryoprotectants,” *PLoS One*, **6**(11).

[12] Marques, L. S., Fossati, A. A. N., Rodrigues, R. B., Da Rosa, H. T., Izaguirry, A. P., Ramalho, J. B., Moreira, J. C. F., Santos, F. W., Zhang, T., and Streit, D. P., 2019, “Slow Freezing versus Vitrification for the Cryopreservation of Zebrafish (Danio Rerio) Ovarian Tissue,” *Sci. Rep.*, **9**(1), pp. 1–11.

[13] Avrami, M., 1939, “Kinetics of Phase Change. I: General Theory,” *J. Chem. Phys.*, **7**(12), pp. 1103–1112.

[14] Avrami, M., 1940, “Kinetics of Phase Change. II Transformation-Time Relations for Random Distribution of Nuclei,” *J. Chem. Phys.*, **8**(2), pp. 212–224.

[15] Avrami, M., 1941, “Granulation, Phase Change, and Microstructure Kinetics of Phase Change. III,” *J. Chem. Phys.*, **9**(2), pp. 177–184.

[16] Zhao, J., Simon, S. L., and McKenna, G. B., 2013, “Using 20-Million-Year-Old Amber to Test the Super-Arrhenius Behaviour of Glass-Forming Systems,” *Nat. Commun.*, **4**, pp. 1–6.

[17] Tucker, M. J., Liebermann, J., 2007, *Vitrification in Assisted Reproduction*, Thomson Publishing Services.

[18] Abidalla, M., and Roversi, P. F., 2018, “Vitrification Assessment: Thermal Analysis of Cryoprotective Aqueous Solutions 1,2 Propanediol and Ethylene Glycol,” *Biopreserv. Biobank.*, **16**(3), pp. 207–216.

[19] Berejnov, V., Husseini, N. S., Alsaied, O. A., and Thorne, R. E., 2006, “Effects of Cryoprotectant Concentration and Cooling Rate on Vitrification of Aqueous Solutions,” *J. Appl. Crystallogr.*, **39**(2), pp. 244–251.

[20] Wowk, B., Fahy, G. M., Ahmedyar, S., Taylor, M. J., and Rabin, Y., 2018, “Vitrification Tendency and Stability of DP6-Based Vitrification Solutions for Complex Tissue Cryopreservation,” *Cryobiology*, **82**, pp.

70–77.

[21] Natesan, H., and Bischof, J. C., 2017, “Multiscale Thermal Property Measurements for Biomedical Applications,” *ACS Biomater. Sci. Eng.*, **3**(11), pp. 2669–2691.

[22] Yi, F., and Lavan, D. A., 2019, “Nanocalorimetry: Exploring Materials Faster and Smaller,” *Appl. Phys. Rev.*, **6**, 031302.

[23] Minakov, A. A., and Schick, C., 2007, “Ultrafast Thermal Processing and Nanocalorimetry at Heating and Cooling Rates up to 1 MKs,” *Rev. Sci. Instrum.*, **78**(7).

[24] Gregson, F. K. A., Ordoubadi, M., Miles, R. E. H., Haddrell, A. E., Barona, D., Lewis, D., Church, T., Vehring, R., and Reid, J. P., 2019, “Studies of Competing Evaporation Rates of Multiple Volatile Components from a Single Binary-Component Aerosol Droplet,” *Phys. Chem. Chem. Phys.*, **21**(19), pp. 9709–9719.

[25] Furuta, T., Sakai, M., Isobe, T., and Nakajima, A., 2009, “Evaporation Behavior of Microliter- and Sub-Nanoliter-Scale Water Droplets on Two Different Fluoroalkylsilane Coatings,” *Langmuir*, **25**(20), pp. 11998–12001.

[26] Kuhn, T., Earle, M. E., Khalizov, A. F., and Sloan, J. J., 2011, “Size Dependence of Volume and Surface Nucleation Rates for Homogeneous Freezing of Supercooled Water Droplets,” *Atmos. Chem. Phys.*, **11**(6), pp. 2853–2861.

[27] Hopkins, J. B., Badeau, R., Warkentin, M., and Thorne, R. E., 2012, “Effect of Common Cryoprotectants on Critical Warming Rates and Ice Formation in Aqueous Solutions,” *Cryobiology*, **65**(3), pp. 169–178.

[28] Song, Y. S., Adler, D., Xu, F., Kayaalp, E., Nureddin, A., Anchan, R. M., Maas, R. L., and Demirci, U., 2010, “Vitrification and Levitation of a Liquid Droplet on Liquid Nitrogen,” *Proc. Natl. Acad. Sci. U. S. A.*, **107**(10), pp. 4596–4600.

[29] Su, F., Zhao, N., Deng, Y., and Ma, H., 2018, “An Ultrafast Vitrification Method for Cell Cryopreservation,” *JOURNAL OF HEAT TRANSFER-TRANSACTIONS OF THE ASME*, **140**(1), pp. 1–4.

[30] Su, F., Fan, Y., Xu, H., Zhao, N., Ji, Y., Deng, Y., and Ma, H., 2020, “Thin-Film Evaporation Heat Transfer of Liquid Nitrogen and Its Application in Cell Vitrification,” *JOURNAL OF HEAT TRANSFER-TRANSACTIONS OF THE ASME*, **142**(7), pp. 1–6.

[31] Daly, J., Zuchowicz, N., Nuñez Lendo, C. I., Khosla, K., Lager, C., Henley, E. M., Bischof, J., Kleinhans, F. W., Lin, C., Peters, E. C., and Hagedorn, M., 2018, "Successful Cryopreservation of Coral Larvae Using Vitrification and Laser Warming," *Sci. Rep.*, **8**(1), pp. 1–10.

[32] Khosla, K., Zhan, L., Bhati, A., Carley-Clopton, A., Hagedorn, M., and Bischof, J., 2019, "Characterization of Laser Gold Nanowarming: A Platform for Millimeter-Scale Cryopreservation," *Langmuir*, **35**(23), pp. 7364–7375.

[33] Warkentin, M., Sethna, J. P., and Thorne, R. E., 2013, "Critical Droplet Theory Explains the Glass Formability of Aqueous Solutions," *Phys. Rev. Lett.*, **110**(1).

[34] Han, Z., and Bischof, J., 2020, "Critical Cooling and Warming Rates as a Function of CPA Concentration," *Cryoletters*, **41**(4), pp. 185–193.

[35] Liu, Y., Kangas, J., Wang, Y., Khosla, K., Pasek-Allen, J., Saunders, A., Oldenburg, S., and Bischof, J., 2020, "Photothermal Conversion of Gold Nanoparticles for Uniform Pulsed Laser Warming of Vitrified Biomaterials," *Nanoscale*, **12**(23), pp. 12346–12356.

[36] Wowk, B., Darwin, M., Harris, S. B., Russell, S. R., and Rasch, C. M., 1999, "Effects of Solute Methoxylation on Glass-Forming Ability and Stability of Vitrification Solutions," *Cryobiology*, **39**(3), pp. 215–227.

[37] Baudot, A., Alger, L., and Boutron, P., 2000, "Glass-Forming Tendency in the System Water-Dimethyl Sulfoxide," *Cryobiology*, **40**(2), pp. 151–158.

[38] Warkentin, M., Stanislavskaia, V., Hammes, K., and Thorne, R. E., 2008, "Cryocrystallography in Capillaries: Critical Glycerol Concentrations and Cooling Rates," *J. Appl. Crystallogr.*, **41**(4), pp. 791–797.

[39] Xue, X., Jin, H. L., He, Z. Z., and Liu, J., 2015, "Quantifying the Growth Rate and Morphology of Ice Crystals Growth in Cryoprotectants via High-Speed Camera and Cryomicroscope," *JOURNAL OF HEAT TRANSFER-TRANSACTIONS OF THE ASME*, **137**(9), pp. 1–5.

[40] Kuleshova, L. L., MacFarlane, D. R., Trounson, A. O., and Shaw, J. M., 1999, "Sugars Exert a Major Influence on the Vitrification Properties of Ethylene Glycol-Based Solutions and Have Low Toxicity to Embryos and Oocytes," *Cryobiology*, **38**(2), pp. 119–130.

[41] Bruggeler, P., and Mayer, E., 1980, "Complete Vitrification in Pure Liquid Water and Dilute Aqueous Solutions," *Nature*, **288**(11), pp. 569–571.

[42] Lankhorst, D., Schriever, J., and Leyte, J. C., 1982, "Determination of the Rotational Correlation Time of Water by Proton NMR Relaxation in $H_2{}^{17}O$ and Some Related Results," *Ber. Bunsenges. Phys. Chem.* **86**, pp. 215–221.

[43] Kuwayama, M., Vajta, G., Kato, O., and Leibo, S. P., 2005, "Highly Efficient Vitrification Method for Cryopreservation of Human Oocytes," *Reprod. Biomed. Online*, **11**(3), pp. 300–308.

[44] Kuwayama, M., 2007, "Highly Efficient Vitrification for Cryopreservation of Human Oocytes and Embryos: The Cryotop Method," *Theriogenology*, **67**(1), pp. 73–80.

[45] Kleinhans, F. W., Seki, S., and Mazur, P., 2010, "Simple, Inexpensive Attainment and Measurement of Very High Cooling and Warming Rates," *Cryobiology*, **61**(2), pp. 231–233.

[46] Welch, A. J., and Van Gemert, M. J. C., 2011, *Optical-Thermal Response of Laser-Irradiated Tissue*, Springer Netherlands.

[47] Wang, T., Zhao, G., Tang, H., and Jiang, Z., 2015, "Determination of Convective Heat Transfer Coefficient at the Outer Surface of a Cryovial Being Plunged Into Liquid Nitrogen," *Cryoletters*, **36**(4), pp. 285–288.

[48] Keblinski, P., Cahill, D. G., Bodapati, A., Sullivan, C. R., and Taton, T. A., 2006, "Limits of Localized Heating by Electromagnetically Excited Nanoparticles," *J. Appl. Phys.*, **100**(5).

[49] Qin, Z., and Bischof, J. C., 2012, "Thermophysical and Biological Responses of Gold Nanoparticle Laser Heating," *Chem. Soc. Rev.*, **41**(3), pp. 1191–1217.

[50] Choi, J. H., and Bischof, J. C., 2008, "A Quantitative Analysis on the Thermal Properties of Phosphate Buffered Saline with Glycerol at Subzero Temperatures," *Int. J. Heat Mass Transf.*, **51**(3–4), pp. 640–649.
