# Supplementary figures and images for: Ultra-Rapid Laser Calorimetry for the Assessment of Crystallization in Low-Concentration Cryoprotectants
Source: J Heat Transfer. 2022 Feb 7;144(3):031207. doi: 10.1115/1.4052568 (PMC8823201; doi:10.1115/1.4052568)

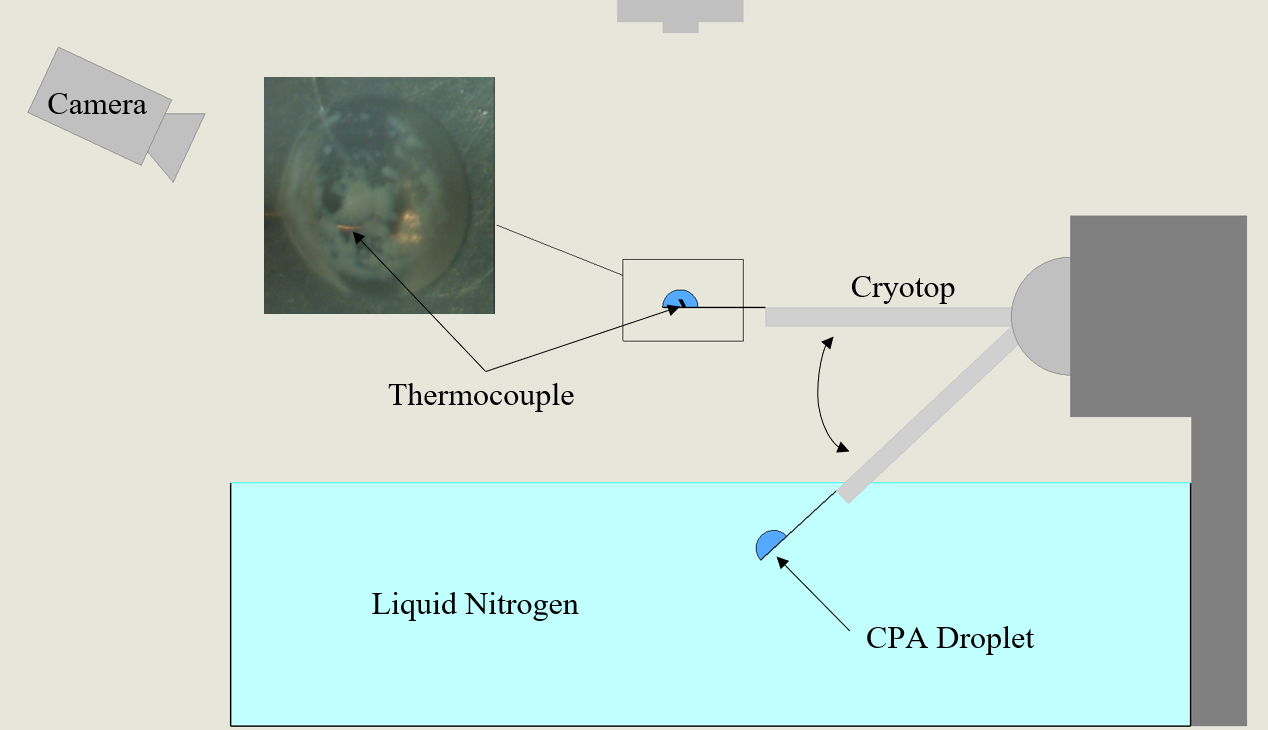

Supplement: Supplementary Material — Supplementary PDF [file Supplementary_Material_HT-21-1359.zip › Supplementary_Material_HT-21-1359_Fig1.tif]

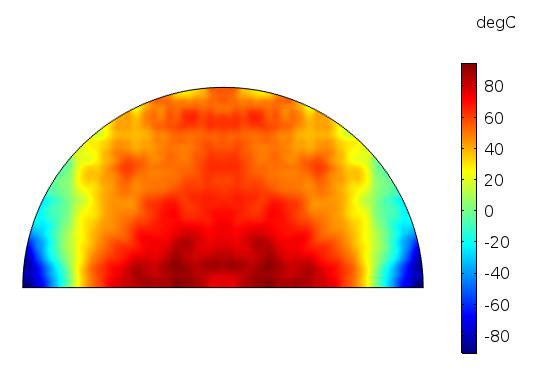

Supplement: Supplementary Material — Supplementary PDF [file Supplementary_Material_HT-21-1359.zip › Supplementary_Material_HT-21-1359_Fig10.tif]

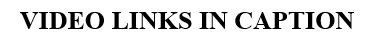

Supplement: Supplementary Material — Supplementary PDF [file Supplementary_Material_HT-21-1359.zip › Supplementary_Material_HT-21-1359_Fig11.tif]

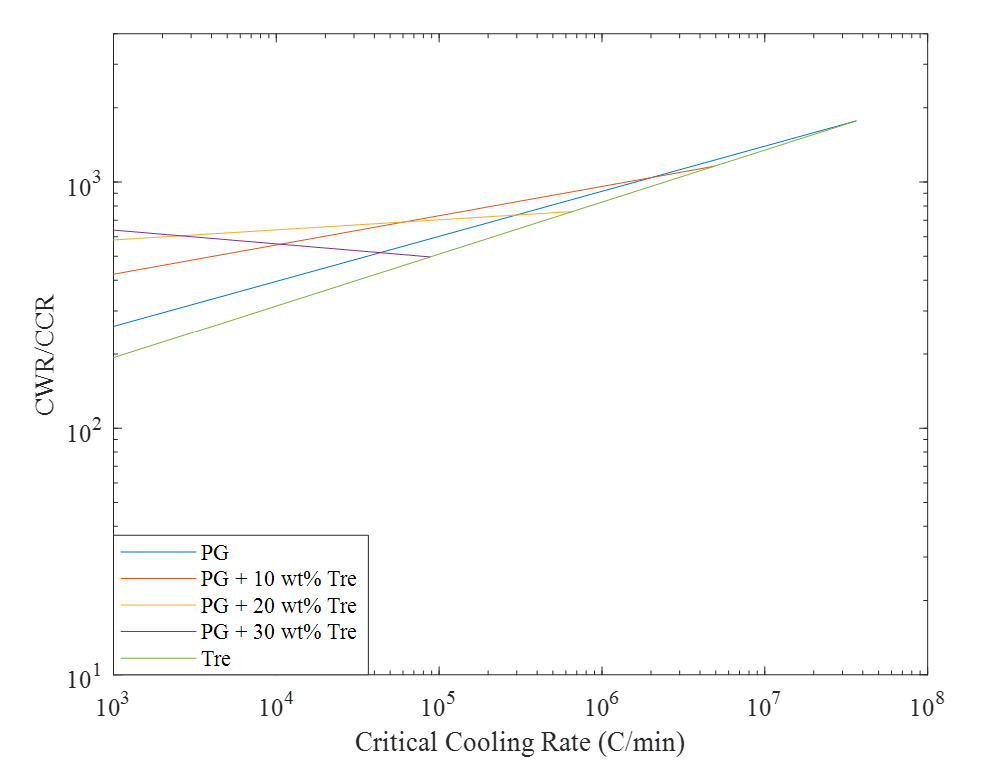

Supplement: Supplementary Material — Supplementary PDF [file Supplementary_Material_HT-21-1359.zip › Supplementary_Material_HT-21-1359_Fig12.tif]

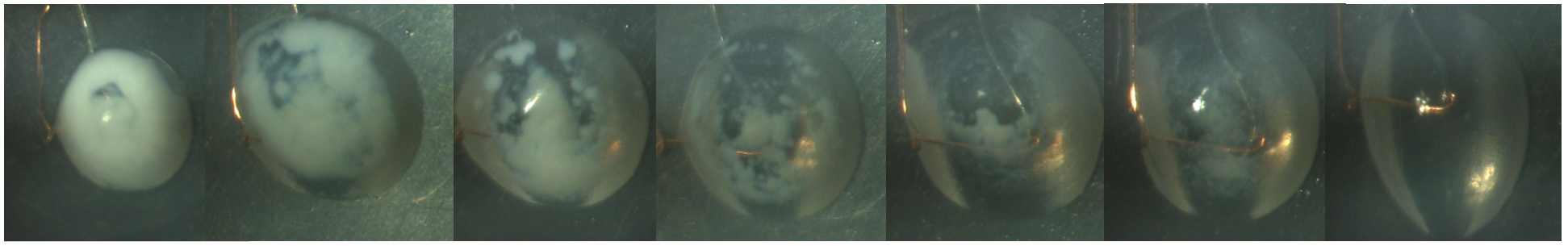

Supplement: Supplementary Material — Supplementary PDF [file Supplementary_Material_HT-21-1359.zip › Supplementary_Material_HT-21-1359_Fig2.tif]

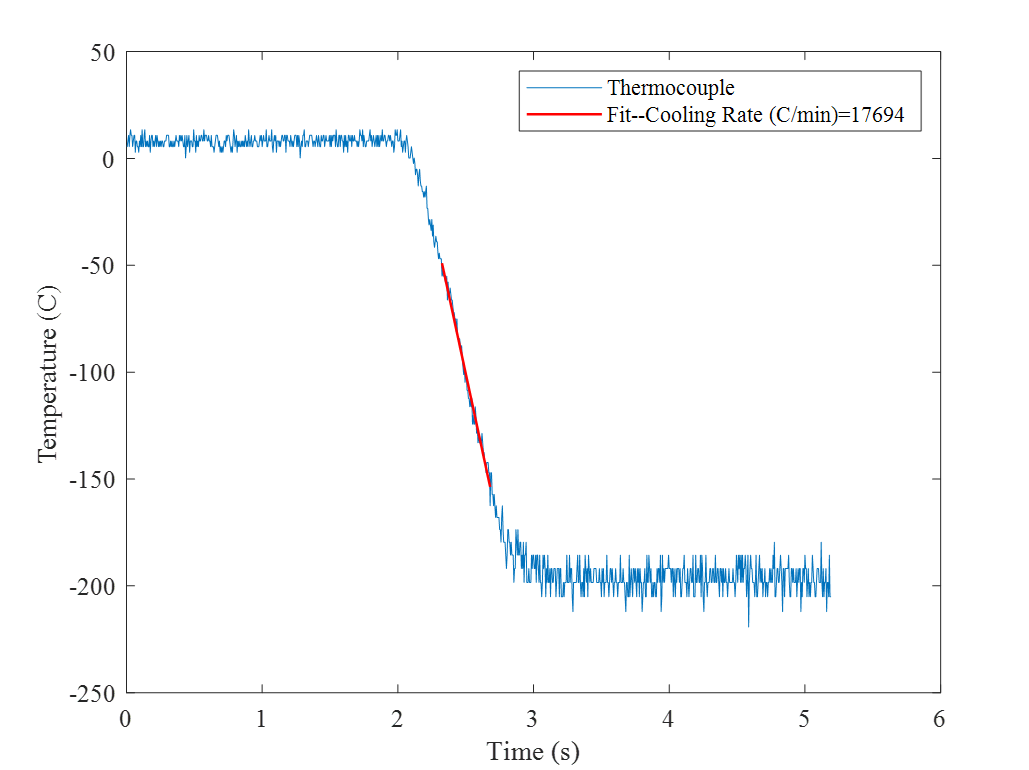

Supplement: Supplementary Material — Supplementary PDF [file Supplementary_Material_HT-21-1359.zip › Supplementary_Material_HT-21-1359_Fig3.tif]

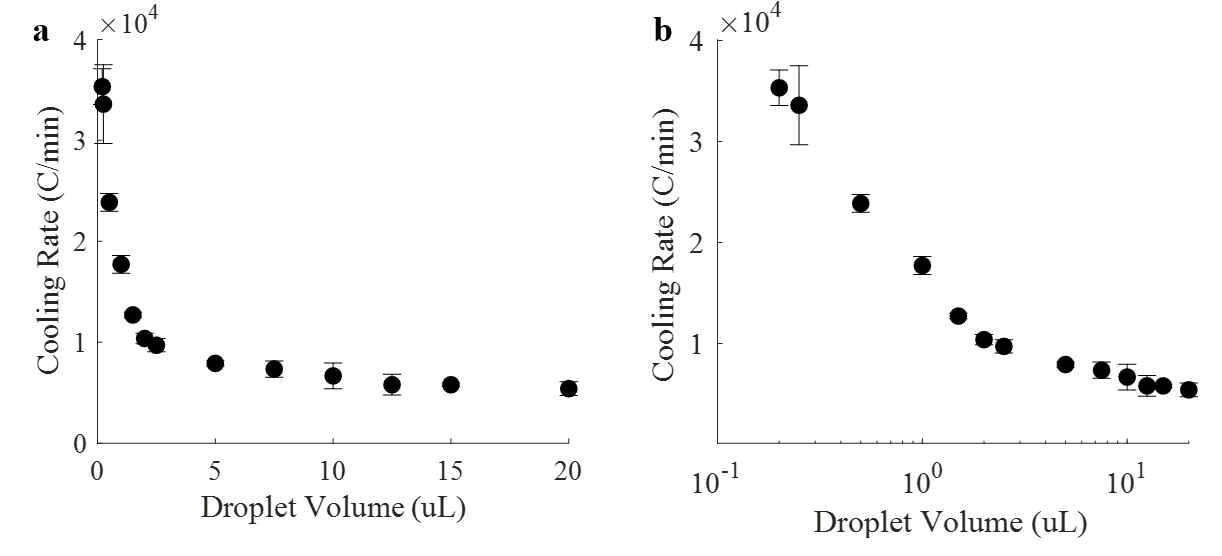

Supplement: Supplementary Material — Supplementary PDF [file Supplementary_Material_HT-21-1359.zip › Supplementary_Material_HT-21-1359_Fig4.tif]

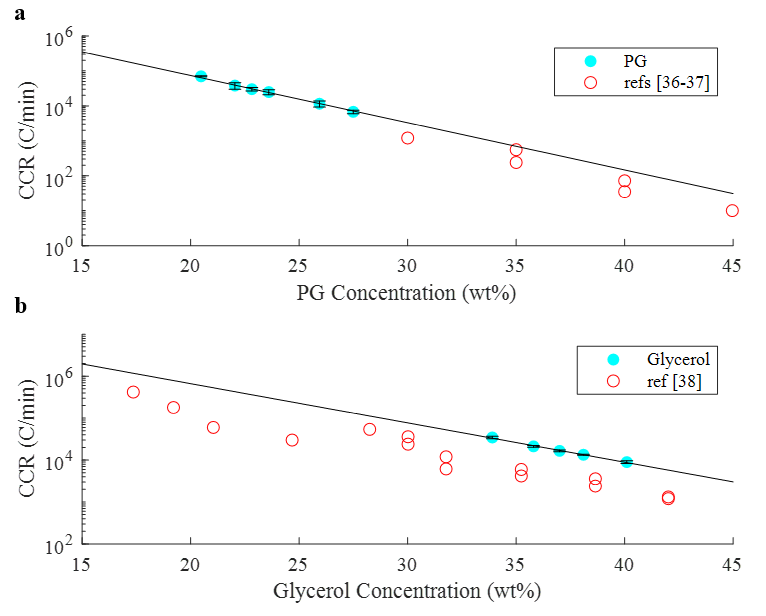

Supplement: Supplementary Material — Supplementary PDF [file Supplementary_Material_HT-21-1359.zip › Supplementary_Material_HT-21-1359_Fig5.tif]

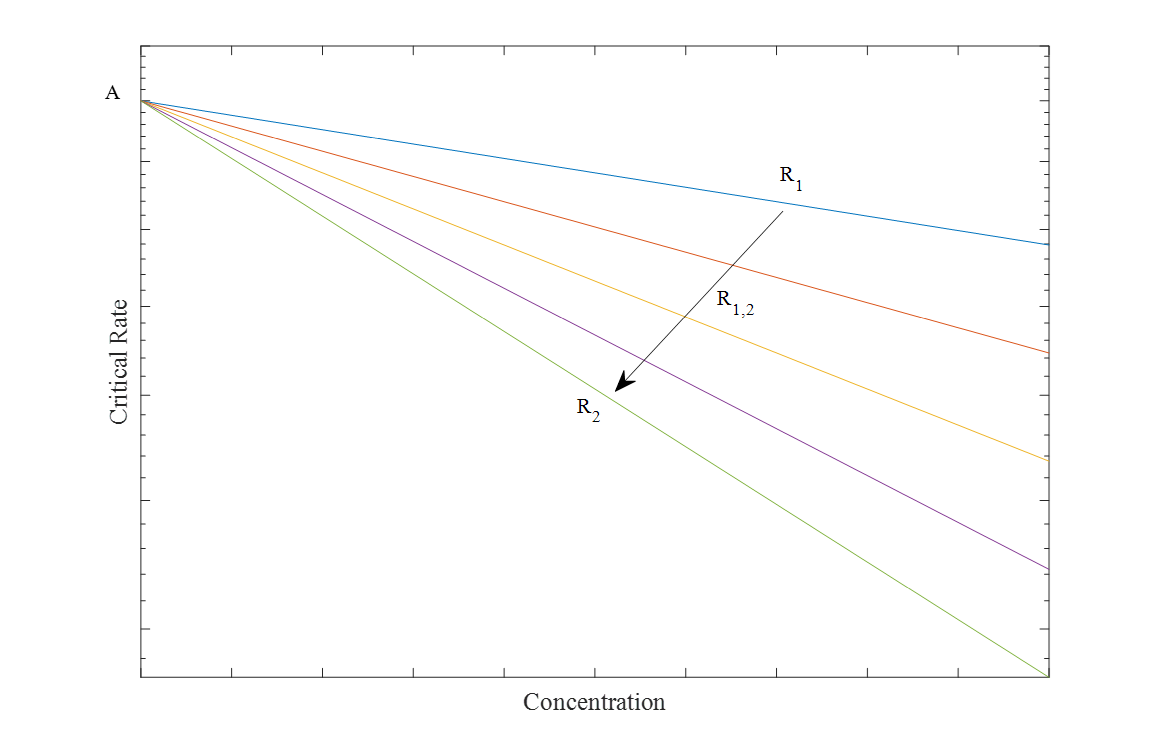

Supplement: Supplementary Material — Supplementary PDF [file Supplementary_Material_HT-21-1359.zip › Supplementary_Material_HT-21-1359_Fig6.tif]

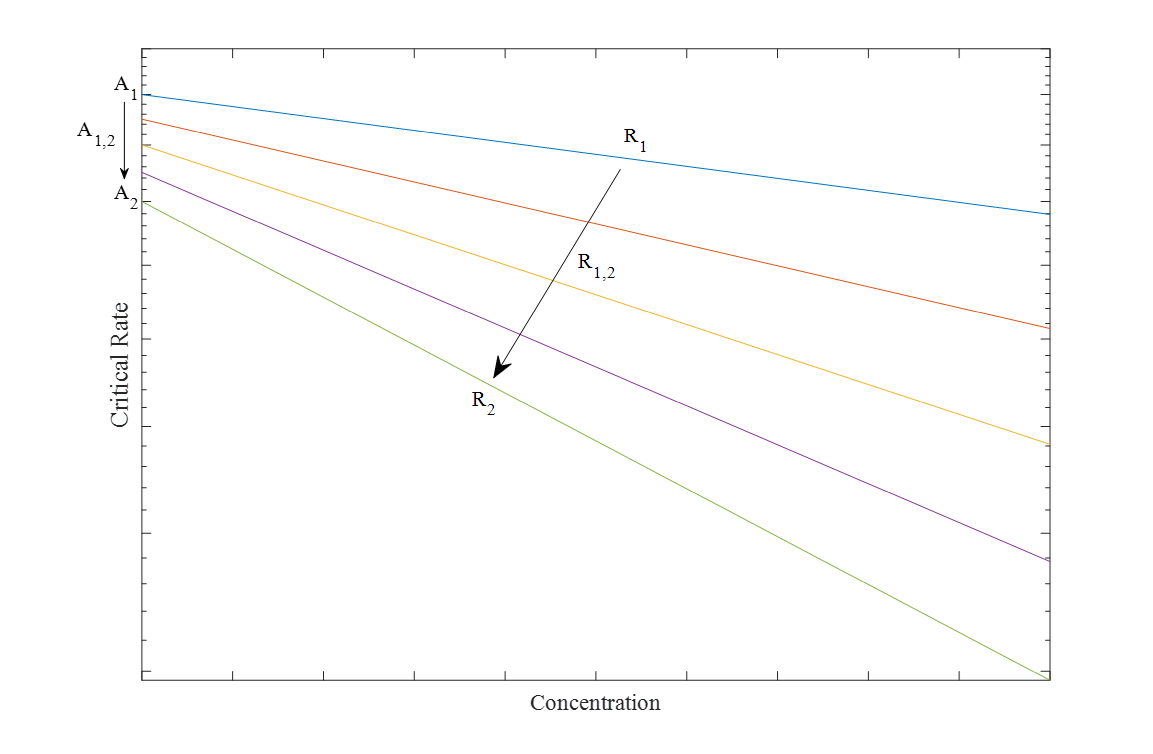

Supplement: Supplementary Material — Supplementary PDF [file Supplementary_Material_HT-21-1359.zip › Supplementary_Material_HT-21-1359_Fig7.tif]

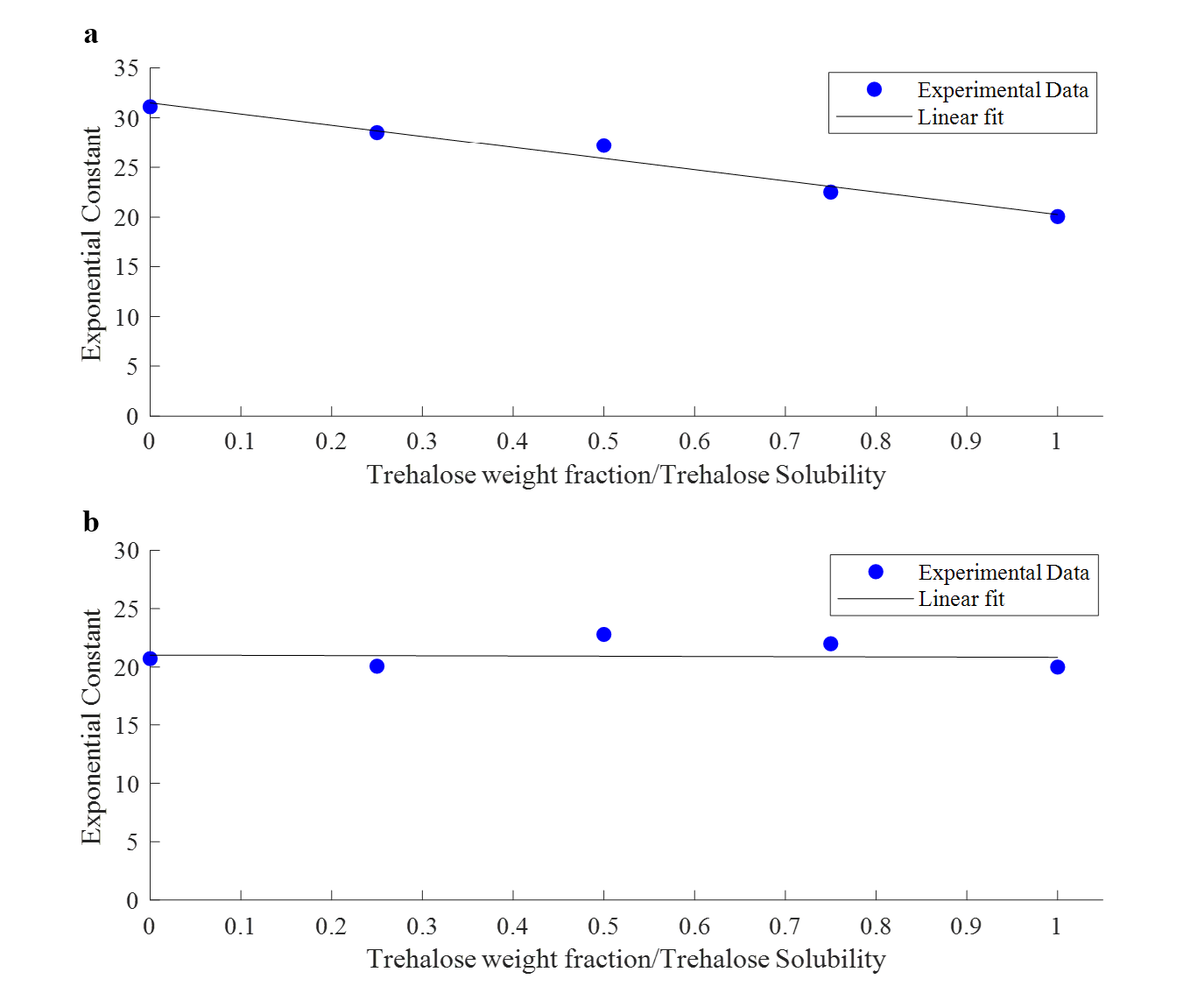

Supplement: Supplementary Material — Supplementary PDF [file Supplementary_Material_HT-21-1359.zip › Supplementary_Material_HT-21-1359_Fig8.tif]

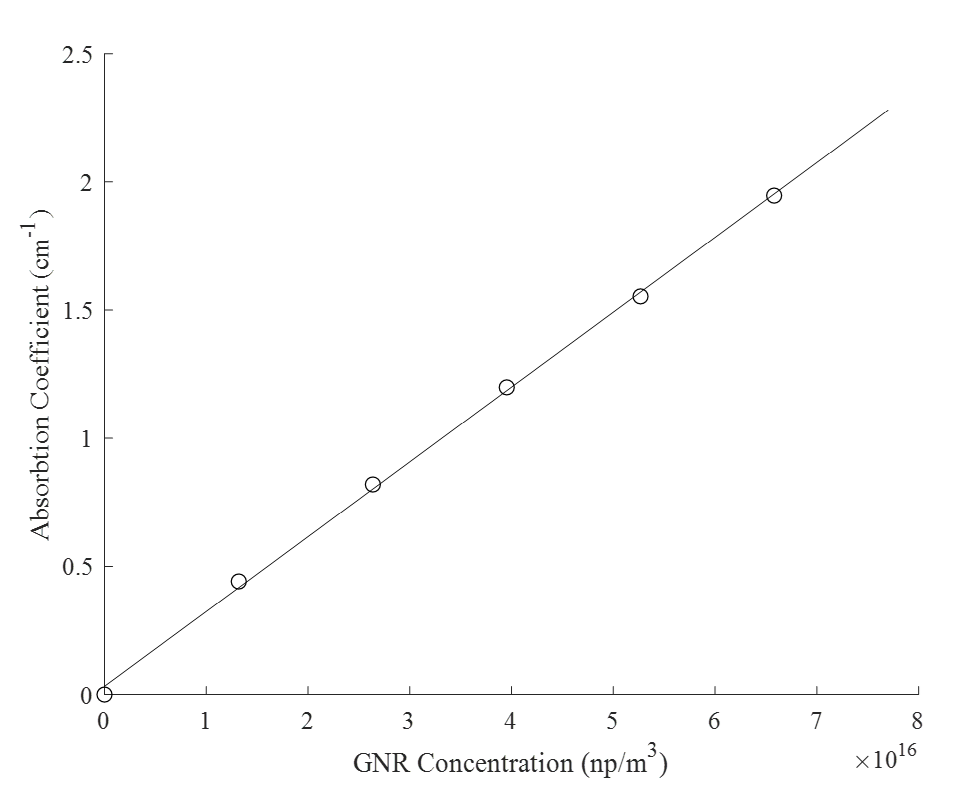

Supplement: Supplementary Material — Supplementary PDF [file Supplementary_Material_HT-21-1359.zip › Supplementary_Material_HT-21-1359_Fig9.tif]
